# CircMETTL6 Suppresses Ovarian Cancer Cell Growth and Metastasis Through Inhibition of GDF15 Transcription by Disrupting the NONO‐POLR2A Complex

**DOI:** 10.1002/advs.202411717

**Published:** 2025-02-03

**Authors:** Mengqian Yu, Mengting Wu, Tao Shen, Qiongzi Qiu, Aoran Luo, Jia Li, Xufan Li, Xiaodong Cheng, Bingjian Lu, Weiguo Lu, Pengyuan Liu, Yan Lu

**Affiliations:** ^1^ Zhejiang Key Laboratory of Precision Diagnosis and Therapy for Major Gynecological Diseases Women's Hospital and Institute of Translational Medicine Zhejiang University School of Medicine Hangzhou Zhejiang 310006 China; ^2^ Department of Gynecologic oncology Women's Hospital Zhejiang University School of Medicine Hangzhou Zhejiang 310006 China; ^3^ Department of Respiratory Medicine Sir Run Run Shaw Hospital and Institute of Translational Medicine Zhejiang University School of Medicine Hangzhou Zhejiang 310016 China; ^4^ Cancer Center Zhejiang University Hangzhou Zhejiang 310013 China; ^5^ Department of Physiology University of Arizona Tucson AZ 85721 USA

**Keywords:** circMETTL6, GDF15, NONO, ovarian cancer, POLR2A, transcription

## Abstract

Circular RNAs (circRNAs) are a distinctive class of non‐coding RNAs with covalent closed‐loop structure, lacking 5′ caps and 3′ poly(A) tails. These molecules are prevalent in eukaryotes and play key roles in cancer. Here, the function of a new circRNA, circMETTL6, in ovarian cancer is identified and investigated. The prognostic significance of circMETTL6 is assessed using RNA in situ hybridization. Functional studies involving circMETTL6 overexpression are performed both in vitro and in vivo. Mechanistic investigations are performed using RNA‐seq, RNA pull‐down, RNA immunoprecipitation, co‐immunoprecipitation, chromatin immunoprecipitation, protein degradation assay and dual‐luciferase reporter assays. circMETTL6 is significantly downregulated in ovarian cancer, and its lower expression correlates with worse prognosis. Overexpression of circMETTL6 significantly inhibited proliferation, migration, and invasion of ovarian cancer cell in vitro, as well as tumor growth and metastasis in vivo. Mechanistically, circMETTL6 recruited the non‐POU domain containing octamer binding protein (NONO) by binding to its Coiled‐coil domain and disrupted its binding with RNA polymerase II subunit A (POLR2A), and consequently inhibiting growth differentiation factor 15 (GDF15) transcription, thereby suppressing ovarian cancer progression. These findings establish circMETTL6 as a novel tumor suppressor in ovarian cancer. Targeting the circMETTL6/NONO/GDF15 axis presents a potential therapeutic avenue for ovarian cancer treatment.

## Graphical abstract

This study identifies a novel circRNA, circMETTL6, downregulated in ovarian cancer, with its reduced expression serving as a prognostic marker for poor outcomes. CircMETTL6 acts as a tumor suppressor by interfering with NONO's interaction with POLR2A, leading to decreased GDF15 transcription. Targeting the circMETTL6/NONO/GDF15 axis may offer valuable insights for prognosis and therapeutic strategies in ovarian cancer.

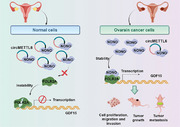

## 1 Introduction

RNA serves as a crucial intermediary in genetic information transfer, with only ≈2% of the genome coding for proteins, the ultimate executors of genetic information and cellular activities.^[^ ^1^, ^2^ ^]^ The majority of RNA molecules are non‐coding RNAs (ncRNAs), which, despite lacking protein‐coding potential, play pivotal roles in regulating various cellular functions. Circular RNAs (circRNAs) represent a novel class of ncRNAs, characterized by their covalently closed‐loop structure formed by back‐splicing of linear pre‐mRNAs.^[^ ^3^, ^4^ ^]^ Due to the absence of 5′ caps and 3′ poly(A) tails, circRNAs are more resistant to exonuclease degradation, resulting in prolonged half‐lives and enhanced stability compared to their linear counterparts.^[^ ^5^ ^]^

CircRNAs have emerged as significant players in cancer diagnosis and therapy, becoming one of the current research hotspots. While numerous studies have highlighted the differential expression of circRNAs and their association with physiological and pathological states,^[^ ^6^ ^]^ their precise molecular mechanisms remain largely underexplored. Beyond their well‐documented role as competing endogenous RNAs (ceRNAs), circRNAs are increasingly recognized for their ability to interact with proteins. This interaction can modulate protein function directly or disrupt protein interactions through recruitment mechanisms. For instance, circ‐Ccnb1 forms a complex with cyclin B1 (CCNB1) and cyclin dependent kinase1 (CDK1) proteins, disrupting the CCNB1‐CDK1 complex and thereby inhibiting cell proliferation, migration, and invasion, and decelerating the cell cycle, ultimately suppressing tumor progression.^[^ ^7^ ^]^ circ‐HuR, which is downregulated in gastric cancer, interacts with CCHC‐type zinc finger nucleic acid binding protein (CNBP) to impede CNBP's binding to the HuR promoter, thus inhibiting cancer cell growth and metastasis.^[^ ^8^ ^]^ circFNDC3B, upregulated in gastric cancer cells, binds to insulin like growth factor 2 mRNA binding protein 3 (IGF2BP3), stabilizing CD44 mRNA and enhancing cell migration and invasion by modulating E‐cadherin expression.^[^ ^9^ ^]^

Ovarian cancer is a highly lethal malignancy of the female reproductive tract with increasing incidence rates annually. According to global cancer statistics for 2020, ovarian cancer was projected to have 313959 new cases and 207252 deaths, rendering it the deadliest cancer of the female reproductive system.^[^ ^10^ ^]^ Due to the lack of specific biomarkers and subtle early‐stage symptoms, most patients are diagnosed at advanced stages with poor prognosis, resulting in a five‐year survival rate below 30%.^[^ ^11^ ^]^ Besides BRCA1/2, there are few effective therapeutic targets, underscoring the urgent need for novel biomarkers and treatment targets in ovarian cancer.^[^ ^12^, ^13^, ^14^ ^]^

In this study, we performed differential expression analysis of circRNAs between ovarian tumors and normal tissues and identified a novel circRNA, circMETTL6, derived from methyltransferase‐like protein 6 (METTL6). circMETTL6 arises from back‐splicing of exons 4–6 of METTL6 pre‐mRNA. METTL6, a tRNA methyltransferase, is known for catalyzing the formation of 3‐methylcytidine at C32 position of specific serine tRNA isoacceptors.^[^ ^15^, ^16^ ^]^ While METTL6 is upregulated in hepatocellular carcinoma (HCC) and promotes tumor progression by affecting cell adhesion molecules,^[^ ^17^ ^]^ we observed that METTL6 itself did not show differential expression in ovarian cancer. Instead, circMETTL6 was significantly downregulated in ovarian cancer tissues and its lower expression was associated with poorer prognosis. Mechanistic studies revealed that circMETTL6 inhibits ovarian cancer progression by recruiting the NONO protein, disrupting its interaction with POLR2A, and subsequently suppressing GDF15 transcription.

## 2 Results

### 2.1 CircMETTL6 is Downregulated in Ovarian Cancer and its Reduced Expression is Associated with Poor Prognosis

**Figure 1:**
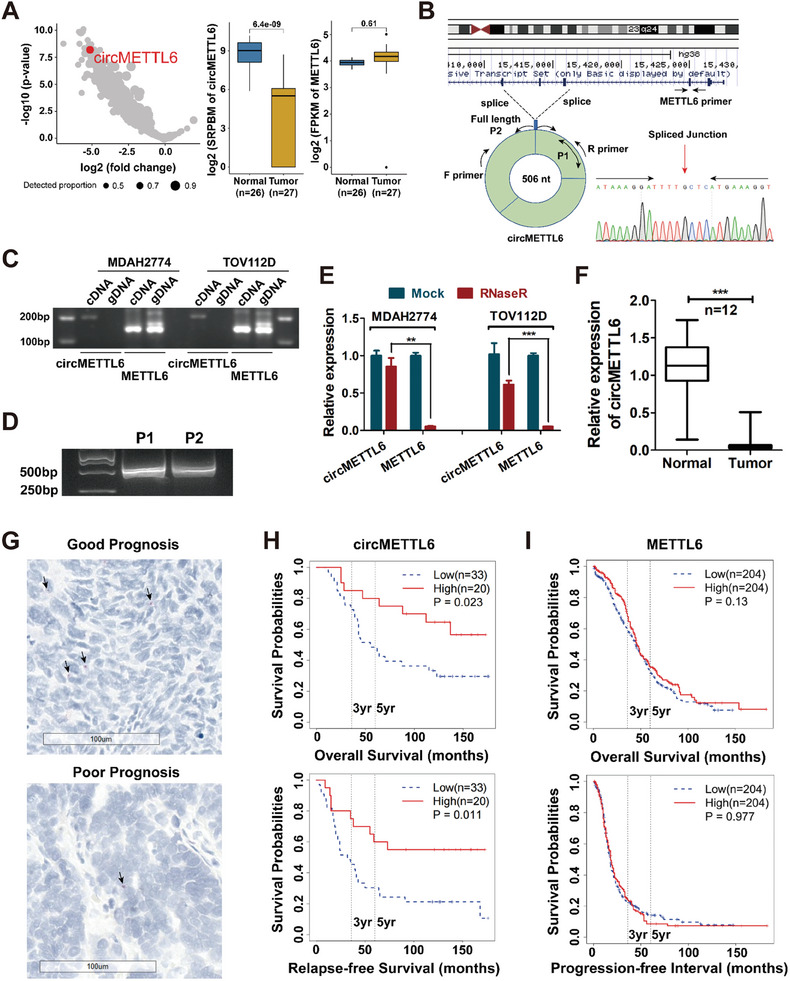
circMETTL6 is downregulated in ovarian cancer. A) Volcano plot depicting the differential expression of circRNAs in ovarian cancer tissues compared to normal ovarian tissues, with circMETTL6 highlighted as a red circle. The right panel shows the expression of circMETTL6 and its parental gene METTL6 in ovarian tumor (*n* = 27) and normal tissue (*n* = 26). Spliced reads per billion mapping (SRPBM) is used to quantify circRNA expression. B) Schematic representation of circMETTL6 formation. circMETTL6 was back‐spliced from exons 4–6 of METTL6, and its full‐length sequence was verified by Sanger sequencing, with spliced junctions depicted. P1 and P2 indicate pairs of full‐length primers used for circMETTL6. C) Agarose gel electrophoresis showing amplification of circMETTL6 using divergent primers in cDNA from MDAH2774 and TOV112D cells, but not in gDNA. D) Amplification of the full‐length circMETTL6 using two different primer sets (P1 and P2). E) qRT‐PCR analysis assessing the relative abundance of circMETTL6 and METTL6 in MDAH2774 and TOV112D cells treated with RNase R. CircMETTL6 exhibited robust exonuclease resistance, while METTL6 did not. F) qRT‐PCR analysis of circMETTL6 expression in an independent cohort consisting of 12 tumor tissues from ovarian cancer patients and normal ovarian tissues from patients with benign gynecological diseases. G) BaseScope images (20× magnification) depicting circMETTL6 expression (indicated by arrow). The upper panel shows high circMETTL6 expressions associated with better prognosis, while the lower panel shows low circMETTL6 expression linked to poor prognosis. H) Kaplan‐Meier survival curves for 53 FFPE tissues of ovarian cancer patients. The BaseScope assay was employed to quantify the expression level of circMETTL6. All histological types of epithelial ovarian cancer were included in the survival analysis. I) Kaplan‐Meier survival curves evaluating linear METTL6 expression in ovarian cancer patients using RNA‐seq data from TCGA (*n* = 408). All histological types of epithelial ovarian cancer were included in the survival analysis. Data were presented as mean ± standard deviation (SD); *n * =  3; Student's two‐sided t‐test was used; NS: no significance, ^*^:*P* < 0.05, ^**^:*P* < 0.01, ^***^:*P* < 0.001. These descriptions are not reiterated in the subsequent figure legends.

To explore the role and mechanism of circRNAs in ovarian cancer, RNA‐seq data from 27 ovarian cancer tissue and 26 ovarian normal tissue were analyzed.^[^ ^18^ ^]^ circMETTL6 was identified as one of the most significantly downregulated circRNAs in ovarian cancer (**Figure** **1**A; Table S1, Supporting Information). CircMETTL6 is spliced from exons 4–6 of the METTL6 gene on the antisense strand of chromosome 3 (from 15452751 to 15457449 bp, hg19 genome build) (Figure S1A, Supporting Information). To confirm the existence of circMETTL6, different primers were designed to amplify the circMETTL6 in ovarian cancer cells (Figure 1B). Agarose gel electrophoresis showed that circMETTL6 was amplified by divergent primers in cDNA but not in genomic DNA (gDNA), whereas METTL6 was amplified in both cDNA and gDNA of MDAH2774 and TOV112D (Figure 1C). Two different full‐length primers were designed and the complete sequence of circMETTL6 was confirmed by sanger sequencing (Figure 1B,D). circMETTL6 contains 506 nucleotides and its full‐length sequence is given in Figure S1B (Supporting Information). Furthermore, circMETTL6 was more resistant to digestion by RNase R exonuclease compared to METTL6 (Figure 1E), indicating its circular structure without 5′ cap and 3′ tail. Quantitative real‐time PCR (qRT‐PCR) analysis of an independent cohort further confirmed downregulation of circMETTL6 in ovarian cancer tissues compared to normal ovarian tissues (Figure 1F). Basescope assay with 1ZZ Basescope probe on formalin‐fixed paraffin‐embedded (FFPE) samples revealed that low circMETTL6 expression positively correlates with poor prognosis in ovarian cancer patients (Figure 1G,H). Interestingly, METTL6 mRNA levels showed no significant difference between ovarian tumors and normal ovarian tissues (Figure 1A; Figure S1C, Supporting Information) and did not correlate with patient prognosis (Figure 1I). Taken together, these findings suggest that circMETTL6 may serve as a potential diagnostic and prognostic biomarker for ovarian cancer.

### 2.2 CircMETTL6 Acts as a Tumor Suppressor in Ovarian Cancer

**Figure 2:**
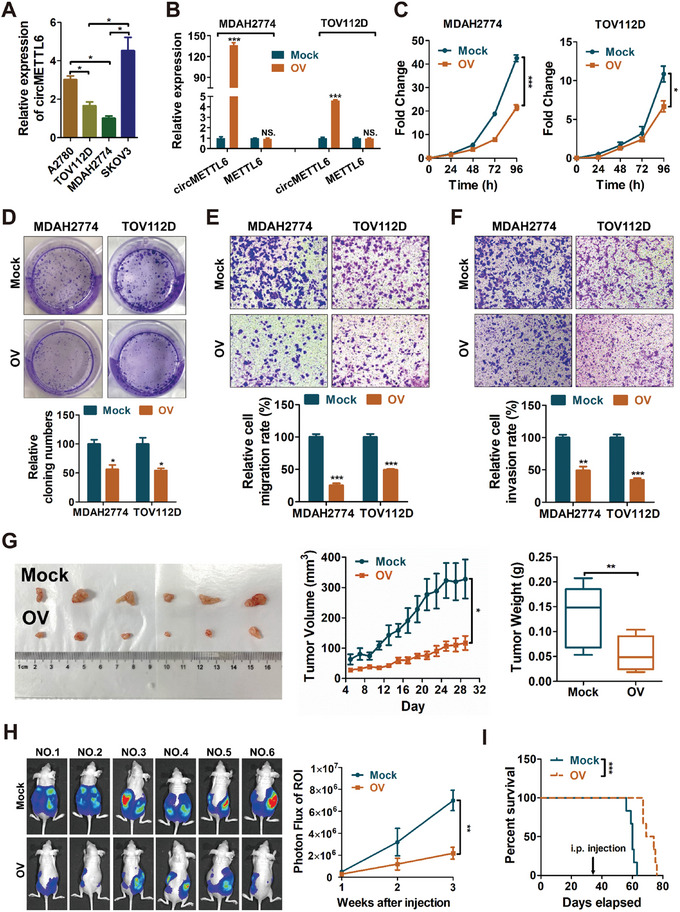
circMETTL6 plays a tumor suppressive role in ovarian cancer cells. A) qRT‐PCR analysis of circMETTL6 expression across various ovarian cancer cell lines. B) qRT‐PCR analysis of circMETTL6 and METTL6 expression in MDAH2774 and TOV112D cells transfected with circMETTL6 (OV) and negative control (Mock) plasmids. C,D) CCK‐8 (C) and colony formation (D) assays in MDAH2774 and TOV112D cells transfected with circMETTL6 or negative control plasmids. E,F) Migration (E) and invasion (F) abilities of MDAH2774 and TOV112D cells transfected with circMETTL6 or negative control plasmids, assessed by transwell assays. Scale bars, 100 µm. Statistical analysis of three independent assays is shown in the lower panel. G) Tumor volume (*middle*) and weight (*right*) of mouse xenograft model injected with circMETTL6 OV and Mock MDAH2774 cells on the right and left flanks (*n* = 6), respectively. Tumor tissues are shown on the left, excised from nude mice 4 weeks post‐inoculation. H) Bioluminescence imaging of metastatic tumor in nude mice injected intraperitoneally with circMETTL6 OV and Mock MDAH2774 cells (*n* = 6). Beginning 7 days after the intraperitoneal injection, bioluminescence imaging was then captured every week. The finally time point images of each mouse are shown in the left panel. Statistical results are shown in the right panel. I) Survival curve of nude mice that had been intraperitoneal injected with circMETTL6 OV and Mock MDAH2774 cells (*n* = 6).

Given the significant downregulation of circMETTL6 in ovarian cancer and its association with poor prognosis, we hypothesized that circMETTL6 may function as a tumor suppressor. To test this, an overexpression vector of circMETTL6 was constructed using the circRNA lentiviral expression vector pLO5. We overexpressed circMETTL6 in MDAH2774 and TOV112D cells, both of which have low endogenous circMETTL6 levels (**Figure** **2**A). The overexpression of circMETTL6 was confirmed via qRT‐PCR, showing a dramatically increase in circMETTL6 levels without altering METTL6 mRNA expression (Figure 2B). Functional assays revealed that circMETTL6 overexpression significantly inhibited cell proliferation, as evidenced by cell counting kit‐8 (CCK8) and colony formation assays (Figure 2C,D). Furthermore, transwell assays showed that circMETTL6 overexpression markedly reduced migration and invasion (Figure 2E,F). In vivo, subcutaneous injection of MDAH2774 cells overexpressing circMETTL6 into nude mice resulted in significantly smaller tumor volumes and weights compared to controls, indicating circMETTL6‐mediated suppression of ovarian cancer growth (Figure 2G). In addition, intraperitoneal injection of these cells showed that circMETTL6 overexpression significantly decreased metastatic ovarian tumor growth and prolonged survival in mice (Figure 2H,I).

To further investigate the role of circMETTL6, we next knocked down its expression in SKOV3 cells, which exhibit high endogenous circMETTL6 levels (Figure 2A), using two siRNAs specifically targeting the junction sites of circMETTL6. CircMETTL6 siRNAs did not alter METTL6 mRNA expression (Figure S2A, Supporting Information). However, depletion of circMETTL6 significantly enhanced ovarian cancer cell proliferation (Figure S2B, Supporting Information), as well as migration and invasion (Figure S2C, Supporting Information). To rule out the potential confounding effects of METLL6 on our results, two siRNAs were designed for targeting METTL6 while avoiding sequences overlapping with circMETTL6. The downregulation of METTL6 had little effect on circMETTL6 expression (Figure S2D,E, Supporting Information), cell growth (Figure S2F, Supporting Information), migration (Figure S2G, Supporting Information), and invasion (Figure S2H, Supporting Information) in ovarian cancer cells.

Collectively, these results indicate that circMETTL6 plays a tumor‐suppressive role in ovarian cancer in vitro and in vivo, which is functionally independent of its parental METTL6 transcript.

### 2.3 CircMETTL6 Physically Interacts with The Oncogenic Protein NONO

**Figure 3:**
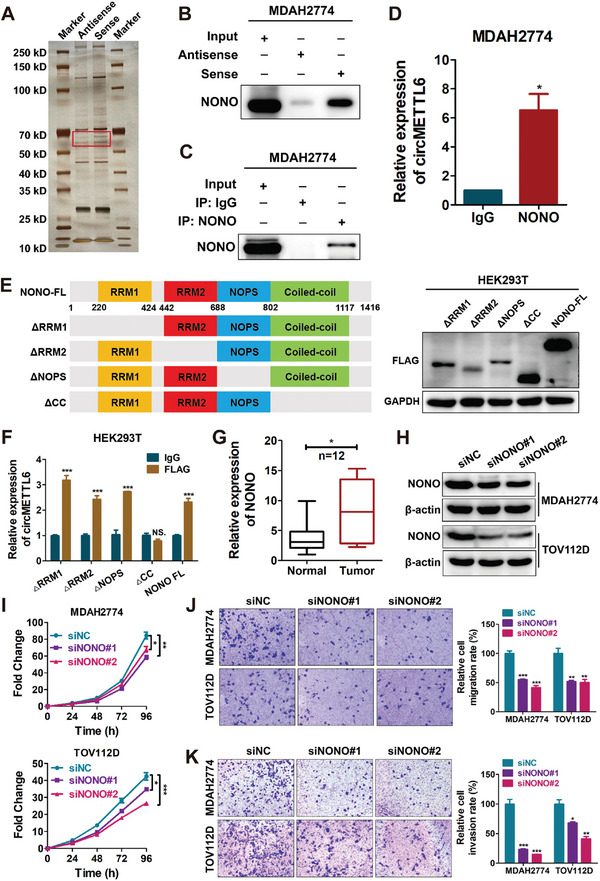
circMETTL6 physically interacts with the oncogenic protein NONO. A) Pull‐down assay in MDAH2774 cells followed by mass spectrometry identifies proteins binding to circMETTL6. An antisense probe was used as a negative control. The distinct band of ≈50–70 kDa is highlighted in the red box. B) RNA pull‐down assay shows interaction between circMETTL6 and NONO, detected by western blotting in the eluate. C) Western blot analysis confirming NONO immunoprecipitation. (D) qRT‐PCR quantification of circMETTL6 enrichment in NONO versus IgG RIP fractions. E) Schematic diagram of the full‐length NONO proteins (FL: 1–1416) and four domain‐deleted mutants of NONO (△RRM1: delete 220–424; △RRM2: delete 442–688; △NOPS: delete 688–802; △CC: delete Coiled‐coil, 802–1117) used in this study (*left*). Western blot analysis of FLAG‐tagged full‐length NONO and its mutants in HEK293T cells (*right*). F) RIP assays with anti‐FLAG antibodies in HEK293T cells transfected with vectors expressing FLAG‐tagged full‐length NONO and four deleted mutants of NONO. IgG was used as negative control group. G) qRT‐PCR analysis of NONO expression in an independent cohort including 12 tumor tissues from ovarian cancer patients and normal ovarian tissues from patients with benign gynecological diseases. H) Western blot analysis of NONO protein expression in ovarian cancer cells transfected with NONO siRNA or control siRNA (siNC). Two siRNAs (siNONO#1 and siNONO#2) were used for targeting NONO. I–K) CCK‐8 (I), migration (J) and invasion (K) assays for MDAH2774 and TOV112D cells transfected with NONO siRNA or control siRNA. For (J,K), statistical analysis is shown in the right panel. Scale bars, 100 µm.

To explore the potential molecular mechanisms of circMETTL6 in ovarian cancer, we designed a specific probe for circMETTL6 and conducted RNA pull‐down assays to identify its binding proteins. Silver staining of the protein obtained from RNA pull‐down revealed a distinct band of ≈50–70 kD in size, which was present in both circMETTL6 sense and antisense samples. This band was further excised from the gel and subjected to mass spectrometry analysis (**Figure** **3**A). Based on the number of distinct peptides and molecular weight (Table S2, Supporting Information), NONO was selected as a specific binding partner of circMETTL6. The association between circMETTL6 and NONO was confirmed by immunoblotting analysis (Figure 3B). To further validate this interaction, RNA immunoprecipitation (RIP) assays were then performed with an anti‐NONO antibody (Figure 3C), which confirmed that NONO bound to circMETTL6 (Figure 3D). NONO comprises four primary structural domains: RNA‐recognition motif1 (RRM1), RRM2, NonA/paraspeckle (NOPS) and Coiled‐coil.^[^ ^19^, ^20^ ^]^ To determine which domain of NONO interacts with circMETTL6, Flag‐tagged full‐length and four domain‐deleted mutants of NONO variants were constructed (Figure 3E). The RIP assay revealed that circMETTL6 predominantly binds to the Coiled‐coil domain of NONO (Figure 3F), indicating that this domain is crucial for the interaction, which is lost upon deletion of the Coiled‐coil domain.

Previous studies have demonstrated that NONO expression is upregulated in various cancers, including breast cancer,^[^ ^21^ ^]^ glioblastoma^[^ ^22^ ^]^ and hepatocellular carcinoma,^[^ ^23^ ^]^ where it promotes tumorigenesis and progression. However, NONO is significantly downregulated in bladder cancer, where it functions to inhibit cancer cell migration and invasion.^[^ ^19^ ^]^ To explore the role of NONO in ovarian cancer, the mRNA and protein expression levels of NONO in ovarian cancer patients were analyzed using our own RNA‐seq data^[^ ^18^ ^]^ and data from the Clinical Proteomic Tumor Analysis Consortium (CPTAC)^[^ ^24^ ^]^ respectively. Our analysis revealed that NONO is markedly overexpressed in ovarian cancer at both mRNA and protein levels (Figure S3A,B, Supporting Information). This upregulation was further confirmed by qRT‐PCR analysis in an independent cohort, comparing ovarian cancer tissues to normal ovarian tissues (Figure 3G). To assess the functional impact of NONO, we knocked down its expression using siRNAs in MDAH2774 and TOV112D ovarian cancer cells (Figure 3H; Figure S3C, Supporting Information). The knockdown of NONO significantly inhibited the proliferation (Figure 3I), migration (Figure 3J) and invasion (Figure 3K) of ovarian cancer cells. Collectively, these findings indicate that circMETTL6 physically interacts with NONO, which plays an oncogenic role by promoting malignant phenotypes in ovarian cancer cells.

### 2.4 CircMETTL6 Binding to NONO Disrupts its Interaction with POLR2A

**Figure 4:**
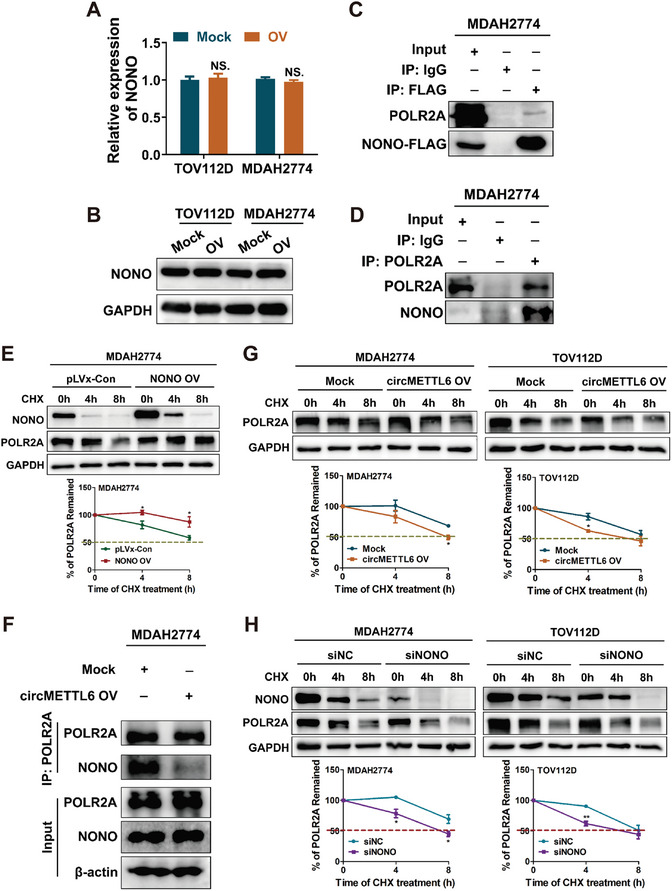
CircMETTL6 binding to NONO disrupts its interaction with POLR2A. A) qRT‐PCR analysis of NONO expression in circMETTL6‐overexpressing ovarian cancer cells. Mock: empty vector. OV: circMETTL6 overexpression. B) Western blot analysis of NONO expression in circMETTL6‐overexpressing ovarian cancer cells. C) Co‐IP assay with Flag antibody and IgG as a negative control reveals the interaction between NONO and POLR2A. D) Co‐IP assay with POLR2A antibody and IgG as a negative control confirms the interaction between NONO and POLR2A. E) Western blot analysis of POLR2A protein level in NONO overexpression (OV) and control (pLVx‐Con) MDAH2774 cells treated with CHX (100 µg mL^−1^) for different durations. Statistical results of remaining POLR2A proteins in three independent replicates are shown in the lower panel. F) Co‐IP assay by POLR2A antibody detecting the interaction between NONO and POLR2A in circMETTL6 OV and Mock MDAH2774 cells. G) Western blot analysis of POLR2A protein level in circMETTL6 OV and Mock ovarian cancer cells (MDAH2774 and TOV112D) treated with CHX (100 µg mL^−1^) for different durations. Statistical results of remaining POLR2A proteins in three independent replicates are shown in the lower panel. H) Western blot analysis of POLR2A protein level in NONO knockdown (siNONO) and control (siNC) ovarian cancer cells (MDAH2774 and TOV112D) treated with CHX (100 µg mL^−1^) for different durations. Statistical results of remaining POLR2A proteins in three independent replicates are shown in the lower panel.

As demonstrated earlier, circMETTL6 and NONO interact with each other, with circMETTL6 acting as a tumor suppressor and NONO as an oncogene in ovarian cancer. To further elucidate the regulatory mechanism between circMETTL6 and NONO, we investigated their interrelationship. Interestingly, circMETTL6 did not affect the expression of NONO at either the mRNA or protein levels (**Figure** **4**A,B). NONO, a multifunctional DNA‐ and RNA‐binding protein, has been implicated in several biological processes through its role in regulating gene transcription and translation.^[^ ^25^, ^26^ ^]^ It is well established that NONO physically binds to the carboxy‐terminal domain (CTD) of the largest subunit of eukaryotic RNA polymerase II (RNA pol II).^[^ ^27^ ^]^ To confirmed this association in ovarian cancer cells, we performed co‐immunoprecipitation (Co‐IP) experiments in MDAH2774 cells. The results validated the interaction between NONO and POLR2A, the largest and catalytically essential subunit of RNA pol II^[^ ^28^ ^]^ (Figure 4C,D). Furthermore, a cycloheximide (CHX) degradation assay revealed that POLR2A protein expression positively correlates with NONO levels, as POLR2A degradation slowed in cells overexpressing NONO (Figure 4E). This finding suggests that NONO stabilizes the POLR2A protein.

To explore the impact of circMETTL6 on this interaction, we conducted Co‐IP experiments in cells overexpressing circMETTL6 compared to control cells. The results showed that circMETTL6 overexpression significantly reduced the binding affinity of NONO to POLR2A (Figure 4F). Importantly, under CHX treatment, the POLR2A protein degradation was markedly accelerated in cells overexpressing circMETTL6 (Figure 4G), similar to the effect observed with NONO knockdown (Figure 4H). Collectively, these results suggested that circMETTL6 disrupts the interaction between NONO and POLR2A, leading to the destabilization of the POLR2A protein.

### 2.5 CircMETTL6 Disrupts NONO‐POLR2A Complex to Suppress GDF15 Transcription

**Figure 5:**
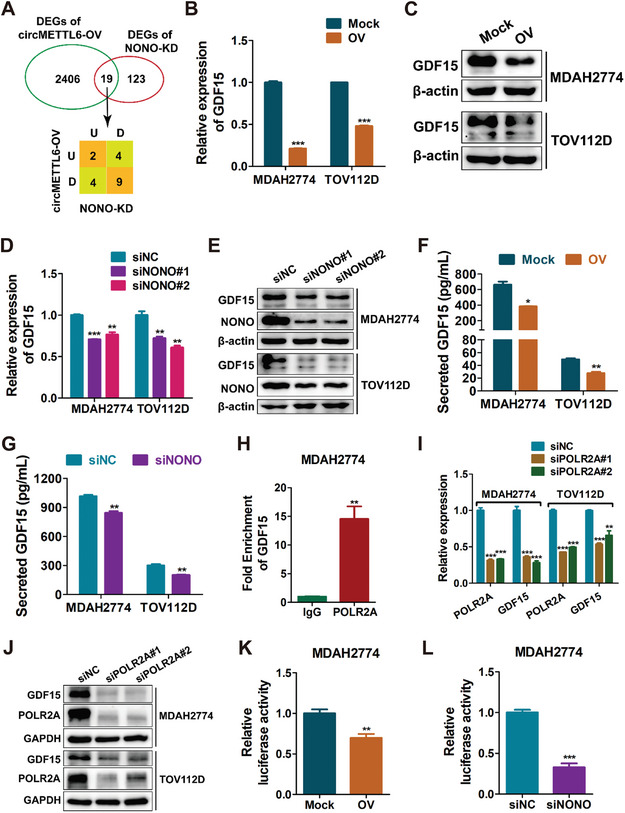
circMETTL6 disrupts NONO‐POLR2A complex to suppress GDF15 transcription. A) Venn diagram of the cross comparisons of two sets of genes: differentially expressed genes (DEGs) from NONO knockdown (KD) and circMETTL6 overexpression (OV) RNA‐seq data. U: up‐regulated; D: down‐regulated. B,C) mRNA (B) and protein (C) expression of GDF15 in MDAH2774 and TOV112D cells with circMETTL6 overexpression, assessed by qRT‐PCR and western blot, respectively. D,E) mRNA (D) and protein (E) expression of GDF15 in MDAH2774 and TOV112D cells transfected with NONO siRNAs or negative control siRNA (siNC), assessed by qRT‐PCR and western blot, respectively. Two siRNAs (siNONO#1 and siNONO#2) were used to target NONO. F,G) ELISA detection of secreted GDF15 protein levels in MDAH2774 and TOV112D cells with circMETTL6 overexpression or mock control (F), or with NONO knockdown or NC (G). H) ChIP‐qPCR analysis of POLR2A binding to the GDF15 promoter in MDAH2774 cells using anti‐IgG or anti‐POLR2A antibody. I,J) mRNA (I) and protein (J) expression of GDF15 in MDAH2774 and TOV112D cells transfected with POLR2A siRNAs or control siRNA (siNC), assessed by qRT‐PCR and western blot, respectively. Two siRNAs (siPOLR2A #1 and siPOLR2A #2) were used to target POLR2A. K) Luciferase reporter assays using pGL3 plasmid with GDF15 promoter sequences in MDAH2774 cells transfected with circMETTL6 or mock plasmids. L) Luciferase reporter assays using pGL3 plasmid with GDF15 promoter sequences in MDAH2774 cells transfected with NONO siRNA or control siRNA.

POLR2A plays an indispensable role in gene expression regulation,^[^ ^28^ ^]^ promoting us to hypothesize that the recruitment of NONO by circMETTL6 may destabilize POLR2A protein and influence the expression of downstream genes. To identify these downstream targets of circMETTL6 and NONO, we performed an unbiased transcriptomic analysis using RNA sequencing (RNA‐seq) in circMETTL6‐overexpressing (Figure S4A, Supporting Information) and NONO‐knockdown TOV112D cells (Figure S4B, Supporting Information) and compared them with their respective control cells. RNA‐seq analyses revealed significant changes in 142 genes following NONO knockdown and 2425 genes upon circMETTL6 overexpression (Adjusted P value <0.05). The Venn diagram revealed 19 genes that were co‐regulated by NONO and circMETTL6 (**Figure** **5**A). Given the opposing roles of circMETTL6 and NONO, we focused on genes that were consistently up‐regulated or down‐regulated in both NONO‐knockdown and circMETTL6‐overexpressing ovarian cancer cells. After intersecting the two RNA‐seq datasets and excluding ncRNAs, we identified ten downstream genes. qRT‐PCR analysis of these ten genes revealed that GDF15 and SAPCD2 had the most consistent and significant expression changes upon circMETTL6 overexpression and NONO knockdown in both MDAH2774 and TOV112D cells (Figure 5B,D; Figure S4C,D, Supporting Information). As anticipated, both circMETTL6 overexpression and NONO knockdown suppressed the mRNA and protein levels of GDF15 in MDAH2774 and TOV112D cells (Figure 5B–E). However, the effect of NONO knockdown on SAPCD2 protein levels was inconsistent between the two cell lines (Figure S4E,F, Supporting Information), narrowing our focus to GDF15.

GDF15, a secreted cytokine, is synthesized as a precursor protein of 308 amino acids in the cytoplasm, containing a signal peptide, propeptide, and mature protein. The precursor forms a disulfide‐linked dimer in the endoplasmic reticulum, which is proteolytically cleaved at the RXXR sequence to produce the mature secreted cytokine GDF15.^[^ ^29^, ^30^, ^31^ ^]^ ELISA assay showed that ovarian cancer cells with circMETTL6 overexpression or NONO knockdown secreted significantly less GDF15 compared to control cells (Figure 5F,G), indicating that circMETTL6 and NONO cooperatively regulate the expression of GDF15. Further, chromatin immunoprecipitation (ChIP)‐qPCR assays revealed that POLR2A is recruited to the GDF15 promoter, but not the SAPCD2 promoter (Figure 5H; Figure S4G, Supporting Information). Additionally, POLR2A knockdown significantly reduced both mRNA and protein expression of GDF15 (Figure 5I,J). GDF15 transcription was similarly reduced by circMETTL6 overexpression (Figure 5K) and NONO knockdown (Figure 5L) in ovarian cancer cells. To further investigate, we truncated the GDF15 promoter sequence into fragments of 1–99, 100–1000, 1–1000, and 1000–2000 bp, and conducted luciferase reporter assays. Knockdown of NONO led to a reduction in GDF15 transcription across all truncations (Figure S4H, Supporting Information) and no common binding motifs were identified between the promoter fragments. This suggests that NONO does not bind directly to the GDF15 promoter but rather interacts with POLR2A, an essential factor for GDF15 transcription. Taken together, these results suggested that circMETTL6 disrupts the NONO‐POLR2A complex, thereby inhibiting POLR2A‐mediated GDF15 transcription. This disruption suppresses GDF15 expression and secretion, highlighting a novel regulatory mechanism in ovarian cancer cells.

### 2.6 CircMETTL6 Inhibits Ovarian Cancer Cells Proliferation and Migration by Downregulating GDF15 Expression

**Figure 6:**
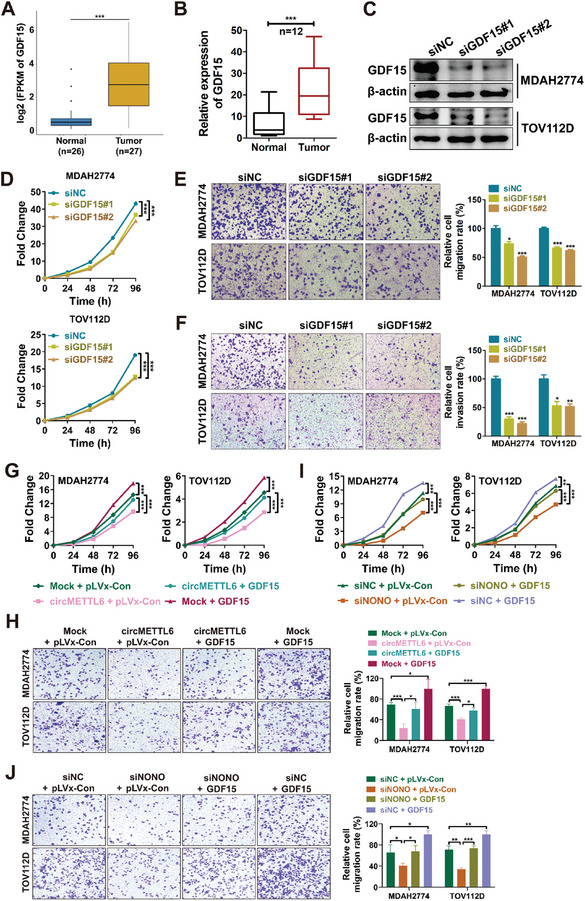
circMETTL6 inhibits ovarian cancer cell proliferation and migration by downregulating GDF15 expression. A) Relative mRNA expression of GDF15 in primary ovarian tumors (*n* = 27) compared to normal ovarian tissues (*n* = 26) as determined by RNA‐seq. B) qRT‐PCR analysis of GDF15 expression in an independent cohort, including 12 ovarian cancer tissues and normal ovarian tissues from patients with benign gynecological diseases. C) Western blot analysis showing GDF15 protein levels in ovarian cancer cells transfected with GDF15 siRNA or control siRNA (siNC). Two siRNAs (siGDF15 #1 and siGDF15 #2) were used for targeting GDF15. D–F) Functional assays assessing the impact of GDF15 knockdown on ovarian cancer cells: CCK‐8 assay (D), migration assay (E) and invasion (F) assay for MDAH2774 and TOV112D cells transfected with GDF15 siRNAs or control siRNA. For (E) and (F), statistical analysis is shown in the right panel. Scale bars, 100 µm. G,H) CCK‐8 (G) and migration (H) assays of circMETTL6‐overexpressing ovarian cancer cells transfected with GDF15 overexpression plasmids or control plasmids (pLVx‐Con). For (H), statistical analysis is shown in the right panel. Scale bars, 100 µm. I,J) CCK‐8 (I) and migration (J) assay for NONO‐silenced ovarian cancer cells transfected with GDF15 overexpression or control plasmids. For (J), statistical analysis is shown in the right panel. Scale bars, 100 µm.

Previous studies have shown that GDF15 is significantly upregulated in cervical cancer, where it promotes cancer cell proliferation by upregulating Cyclin D1 and Cyclin E1 while downregulating p21 through PI3K/AKT and MAPK/ERK signaling pathways.^[^ ^32^ ^]^ Elevated plasma GDF15 levels also serve as a biomarker for colorectal cancer and are associated with increased risk of recurrence.^[^ ^33^ ^]^ Similarly, high plasma GDF15 levels in endometrial cancer patients are linked to lymph node metastasis and poor survival.^[^ ^34^ ^]^ In our RNA‐seq data,^[^ ^18^ ^]^ GDF15 was significantly upregulated in ovarian cancer tissues compared to normal tissues (**Figure** **6**A), confirmed by qRT‐PCR analysis in an independent cohort (Figure 6B). To further investigate the role of GDF15 in ovarian cancer, we designed specific siRNAs to knock down GDF15 in MDAH2774 and TOV112D ovarian cancer cells. qRT‐PCR and western blot analyses confirmed effective reduction of GDF15 mRNA and protein levels by siRNAs (Figure 6C; Figure S5A, Supporting Information). CCK8 assays revealed that GDF15 knockdown significantly impaired the proliferation of ovarian cancer cells (Figure 6D), while transwell assays showed that GDF15 knockdown inhibited cell migration and invasion in both MDAH2774 and TOV112D cells (Figure 6E,F). These findings indicate that GDF15 plays a key role in promoting ovarian cancer cell proliferation and migration. To investigate whether circMETTL6 mediate the function of GDF15 in ovarian cancer cells, GDF15 was overexpressed in circMETTL6‐overexpressing cells via lentiviral transfection (Figure S5B, Supporting Information). GDF15 overexpression promoted the proliferation (Figure 6G,I) and migration (Figure 6H,J) in both MDAH2774 and TOV112D cells, and could rescue the reduced proliferation (Figure 6G) and migration (Figure 6H) observed in circMETTL6‐overexpressing cells. Similarity, the role of NONO in regulating the function of GDF15 was explored, and the results showed that GDF15 overexpression rescued the decreases in proliferation (Figure 6I) and migration (Figure 6J) caused by NONO knockdown (Figure S5C, Supporting Information). These results suggested that circMETTL6 suppresses ovarian cancer cell proliferation and migration by inhibiting NONO‐POLR2A‐mediated GDF15 transcription.

## 3 Discussion

**Figure 7:**
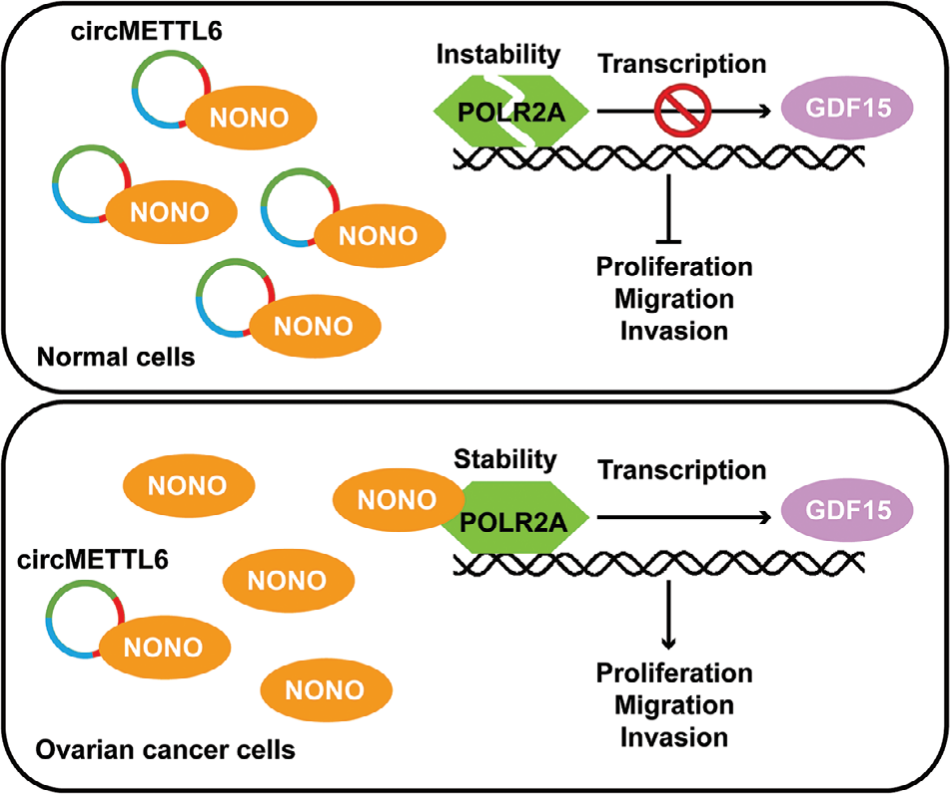
Schematic diagram of circMETTL6‐mediated suppression of ovarian cancer growth and progression. Under normal physiological conditions, upregulated circMETTL6 binds to the oncogenic NONO, disrupting the NONO‐POLR2A complex. This disruption leads to the destabilization of POLR2A, which in turn inhibits the transcription of GDF15, maintaining its expression and secretion at low levels in normal cells. In contrast, in ovarian cancer cells, less circMETTL6 binding to NONO stabilizes POLR2A, promoting increased transcription of GDF15. This elevated GDF15 expression contributes to enhanced tumor cell proliferation, migration and invasion.

Ovarian cancer is a malignancy with a high fatality‐to‐case ratio, primarily due to the lack of effective tumor biomarkers for early detection. Sequencing studies have revealed numerous differentially expressed circRNAs in ovarian cancer tissues, serum and plasma samples.^[^ ^35^, ^36^ ^]^ However, the functional roles of circRNAs in ovarian cancer remain largely unexplored. Our study highlights a novel circRNA, circMETTL6, which was downregulated in ovarian cancer tissues. Notably, lower circMETTL6 expression correlates with poorer prognosis in patients. Functionally, circMETTL6 overexpression inhibits ovarian cancer cell proliferation, migration, and invasion both in vitro and in vivo. Mechanistically, circMETTL6 exerts its effects by binding to the coiled‐coil domain of NONO, leading to the disruption of the NONO‐POLR2A complex. This disruption destabilizes the POLR2A protein, resulting in the inhibition of GDF15 transcription and a significant reduction in GDF15 expression and secretion in ovarian cancer cells. Restoration of GDF15 expression can rescue the inhibited cell proliferation and metastasis caused by circMETTL6 overexpression, underscoring the critical role of circMETTL6 in disrupting NONO/POLR2A‐mediated GDF15 upregulation and subsequent ovarian cancer progression (**Figure** **7**).

NONO is a multifunctional DNA‐ and RNA‐binding protein involved in various aspects of RNA metabolism, including splicing,^[^ ^37^, ^38^ ^]^ transcription,^[^ ^25^, ^39^ ^]^ and DNA repair.^[^ ^40^ ^]^ However, our results showed that NONO does not bind directly to the GDF15 promoter (Figure S4H, Supporting Information) but interacts with POLR2A, a key factor for GDF15 transcription. NONO stabilizes POLR2A protein, while circMETTL6 overexpression weakens this interaction, leading to POLR2A destabilization (Figure 4E–H). Nonetheless, overexpression of circMETTL6 did not completely destroy POLR2A protein stabilization. This indicates that POLR2A stabilization is not solely dependent on NONO. The phosphorylation of the CTD of POLR2A is also important for the coordination of transcription events.^[^ ^41^ ^]^ Nevertheless, circMETTL6 overexpression sufficiently prevents GDF15 transcription in a POLR2A‐dependent manner, and GDF15 overexpression can counteract the inhibitory effects of circMETTL6 on ovarian cancer cell proliferation and metastasis. These findings suggest that NONO/POLR2A‐mediated GDF15 transcription is crucial for ovarian cancer growth and progression, and circMETTL6 functions as a tumor suppressor by disrupting this process.

GDF15, a member of the transforming growth factor‐β superfamily, has context‐dependent roles in cancer.^[^ ^42^ ^]^ Our study demonstrates that GDF15 promotes ovarian cancer cell proliferation, migration, and invasion. Additionally, we show that GDF15 overexpression can reverse the inhibitory effects of circMETTL6 overexpression or NONO knockdown on ovarian cancer cell proliferation and migration. Our results indicate that circMETTL6 and NONO not only regulate GDF15 expression at both mRNA and protein levels but also impact GDF15 secretion. These findings reveal a previously unrecognized regulatory mechanism of GDF15 and its carcinogenic role in ovarian cancer. However, further investigation is needed to elucidate the specific molecular mechanisms through which GDF15 promotes ovarian cancer development.

## 4 Conclusion

In summary, our study identifies circMETTL6 as a novel circRNA downregulated in ovarian cancer, with its reduced expression serving as a prognostic marker for poor outcomes. CircMETTL6 acts as a tumor suppressor by interfering with NONO's interaction with POLR2A, leading to decreased GDF15 transcription. Targeting the circMETTL6/NONO/GDF15 axis may offer valuable insights for prognosis and therapeutic strategies in ovarian cancer.

## 5 Experimental Section

### Identification of circRNAs

The RNA‐seq data of 27 ovarian cancer tissues and 26 normal ovarian tissues that were collected from the Women's Hospital of Zhejiang University (Hangzhou, China) were obtained from our previous work.^[^ ^18^ ^]^ Briefly, the RNA‐seq sequence reads were pre‐processed using Trim Galore, with low‐quality sequences (base quality <20) trimmed from both ends. The trimmed sequence reads were then aligned to the human genome and gene annotation (hg19) using TopHat2 (v 2.0.13).^[^ ^43^ ^]^ Unmapped sequence reads were further analyzed for circRNA identification using CIRC‐explorer2.^[^ ^44^ ^]^ Differential expression of circRNAs between ovarian cancer and normal tissues was assessed using two‐tailed Student's t‐tests. The top 10 differentially expressed circRNAs (Table S1, Supporting Information), which showed no significant differences in their corresponding parental mRNA expression, were selected for subsequent validations. These circRNAs were amplified by PCR using divergent primers in ovarian cancer cell lines to verify the authenticity of their endogenous presence.

### Cell Culture

Human ovarian cancer cells (TOV112D, MDAH2774, and SKOV3) and HEK293T cells were obtained from the American Type Culture Collection (ATCC, Manassas, VA, USA). TOV112D, MDAH2774, and HEK293T cells were cultured in DMEM media (Gibco, Carlsbad, CA, USA) supplemented with 10% FBS, penicillin (100 U mL^−1^), and streptomycin (100 ng mL^−1^) in a 5% CO_2_ incubator at 37 °C. SKOV3 cells were cultured in McCoy's 5A media (Gibco) supplemented with 10% FBS, penicillin (100 U mL^−1^) and streptomycin (100 ng mL^−1^) in a 5% CO_2_ incubator at 37 °C.

### Cell Transfection

The pLO5‐ciR control vector was purchased from GENESEED (Guangzhou, China). To construct the pLO5‐circMETTL6 overexpression vector, the exons 4–6 of METTL6 on the antisense strand of chromosome 3 were inserted into the pLO5‐ciR vector. Cells were infected with pLO5‐circMETTL6 or pLO5‐ciR lentivirus produced in HEK293T cells. Small interference RNAs (siRNAs) targeting NONO, POLR2A, and GDF15 (Table S3, Supporting Information) were designed and synthesized by GenePharma (Shanghai, China). Transfection was performed using GeneMute™ reagent (SignaGen Laboratories, Rockville, MD, USA).

### CCK‐8 Assay and Colony Formation

Equal number of cells were seeded into 96‐well plates with 5 replicates per condition, according to the characteristics of the cells. Cell viability was measured using the CCK‐8 assay (Dojindo Laboratories, Kumamoto, Japan) at various time points by measuring absorbance at 450 nm. For colony formation assay, 2000 cells per well were seeded into 12‐well plates. After one week, colonies were fixed with methanol and stained with 0.1% crystal violet.

### Cell Migration and Invasion Assay

Cell migration and invasion were assessed using a 24‐well Transwell® system with 8.0 µm pore polyester membrane inserts (Corning, Costar, Tewksbury, MA, USA). TOV112D and MDAH2774 cells transfected with siRNAs or stably transfected with pLO5‐circMETTL6 or pLO5‐ciR were seeded into 6‐well plates for 24 h, and then cultured with serum‐free medium for cell starvation. The starved cells were plated with 300 µL cell suspension (3×10^4^ cells) into the upper chamber of the Transwell (migration assay: non‐coated membrane; invasion assay: matrigel‐coated membrane). The lower chamber contained 500 µL medium with 10% FBS. After 24 h, cells were fixed with methanol and stained with 0.1% crystal violet. Images were captured using Leica DM4000 microscope (Bufalo Grove, IL, USA), and each sample was randomly photographed with 3 fields of view at 10× microscope objective and the average value was calculated to minimize bias. Three independent experiments were performed for each condition.

### Quantitative Real‐Time PCR (qRT‐PCR) Analysis

Total RNA was extracted with Trizol (Invitrogen, CA, USA), followed by reverse transcription of 1 µg total RNA into cDNA using HiScript II Q RT SuperMix for qPCR (+ gDNA wiper) (Vazyme, Nanjing, China) in a 20 µL reaction volume. qRT‐PCR was conducted in a 10 µL reaction volume containing SYBR Green PCR Master Mix (Vazyme), 1 µL cDNA, and amplification primers (Table S3, Supporting Information). Relative expression levels were calculated using the 2^−△△CT^ method.

### Tissue Validation

Tissue validation for circMETTL6, METTL6, NONO, and GDF15 expression was performed using qRT‐PCR in an independent cohort comprising tumor tissues from 12 ovarian cancer patients and normal ovarian tissues from 12 patients with benign gynecological diseases. Tissue samples were obtained from the tissue bank of Women's Hospital of Zhejiang University (Hangzhou, China). The diagnosis of all tissues was confirmed histopathologically with TNM clinical stages, following the criteria established by the American Joint Committee on Cancer and the Union for International Cancer Control (2002). All tissue samples used in the current study were collected with informed consent at the time of diagnosis, prior to any treatment. The study protocol was reviewed and approved by the respective Institutional Review Boards of the hospital. The demographic and clinical characteristics of ovarian cancer patients used in qRT‐PCR assays are listed in Table S4 (Supporting Information).

### Western Blot

Cells were lysed with RIPA buffer (Beyotime, Shanghai, China) containing phenylmethanesulfonyl fluoride (PMSF, Beyotime). Lysates were centrifuged at 14000 rpm for 30 min at 4 °C. Protein concentration was determined using a Pierce™ BCA Protein Assay Kit (Thermo Fisher Scientific, Waltham, MA, USA). Equal amounts of proteins were separated by SDS‐PAGE gel and transferred to a polyvinylidene fluoride (PVDF) membrane (Millipore, Burlington, MA, USA). Membranes were blocked with 5% skim milk for at least one hour, then incubated with primary antibodies overnight at 4 °C. Next, the membranes were incubated with an horseradish peroxidase (HRP)‐linked antibody at a 1:2000 dilution for 1 h at room temperature. Proteins were detected using Meilunbio® FGSuper Sensitive ECL Luminescence Reagent (Meilunbio, Dalian, China). Antibody details are listed in Table S5 (Supporting Information).

### Basescope Assays

A total of 53 FFPE tissues of ovarian cancer were obtained from the tissue bank of Women's Hospital of Zhejiang University. Basescope assays utilize z‐probes to enhance detection sensitivity and reduce background noise.^[^ ^45^, ^46^ ^]^ A 1ZZ Basescope probe targeting the junction sequences of circMETTL6 was designed (Advanced Cell Diagnostics, Newark, CA, USA). BaseScope assays were performed on FFPE tissues from ovarian cancer patients using a BaseScope Reagent Kit V2‐RED (Advanced Cell Diagnostics, Cat# 323 900) following the manufacturer's instructions. At 20× magnification, red dots were counted in 10 randomly selected regions per image to quantify circMETTL6 expression. The demographic and clinical characteristics of these ovarian cancer patients used in Basescope assays are listed in Table S6 (Supporting Information).

### Animal Studies

All animal procedures were approved by in Zhejiang University accordance with the NIH Guide for the Care and Use of Laboratory Animals. To establish xenograft tumor model, MDAH2774 cells with stable transfection of circMETTL6 overexpression or control vector (8 × 10^6^ cells) were suspended in 0.1 mL PBS and subcutaneously inoculated into either flank of five‐week‐old female BALB/c nude mice (6 mice per group). Tumor growth was monitored every 2 days for 4 weeks, and tumor volumes were calculated using the following formula: Volume (cm^3^) = (length × width^2^)/2. After four weeks, mice were euthanized, and tumors were excised and measured.

For metastasis studies, MDAH2774 cells with stable transfection of circMETTL6 overexpression or control vector (2 × 10^6^ cells) were injected intraperitoneally into five‐week‐old female BALB/c nude mice (6 mice per group). Mice were administered 150 ng luciferin (Gold Biotech, MO, USA) per gram body weight intraperitoneally. Metastases were monitored using an IVIS@ Lumina II system (Caliper Life Sciences, MA, USA) and bioluminescence imaging weekly for three weeks. Animal survival data were analyzed by log‐rank test.

### Analysis of RNA‐seq Data for Cells with circMETTL6 Overexpression and NONO Knockdown

Total RNA was extracted from TOV112D cells stably transfected with circMETTL6 overexpression plasmid or control plasmid, and from TOV112D cells transfected with either NONO siRNAs or negative control siRNA. RNA‐seq libraries were prepared using Illumina's TruSeq RNA Sample Preparation Kit, as described previously.^[^ ^47^ ^]^ Sequence data were preprocessed and aligned to the human genome (hg19) using TopHat2 (v 2.0.13) as mentioned earlier.^[^ ^43^ ^]^ Transcripts were assembled and identified using Stringtie2 (v2.1.0).^[^ ^48^ ^]^ Gene expression levels were quantified as fragments per kilobase of transcript per million (FPKM) and log2‐transformed for normalization prior to analysis. Differential expression analysis was performed using DEseq2, with differentially expressed genes (DEGs) detected based on adjusted P‐value < 0.05.^[^ ^49^ ^]^

### RNA Pull‑Down Assay

RNA pull‐down assays were performed using the Pierce™ Magnetic RNA‐Protein Pull‐Down Kit (Thermo Fisher Scientific). Biotin‐labeled circMETTL6 probes and antisense probes were synthesized by RIBOBIO (Guangzhou, China). MDAH2774 cells were lysed in IP Lysis Buffer for 30 min, followed by centrifugation at 14000 rpm for 30 min at 4 °C to ensure a protein concentration > 2 mg mL^−1^ in the cell lysate. The resulting lysate fractions were evenly divided into 2 parts. Subsequently, 100 pmol of antisense or sense probes were incubated with magnetic beads and RNA Capture Buffer for 1 hour at room temperature. Equal amounts of lysate were then incubated with probe‐beads complex overnight at 4 °C. After RNA pull‐down, proteins were separated by SDS‐PAGE and visualized with silver staining. Distinct bands between circMETTL6 sense and antisense were excised for mass spectrometry analysis using an Ultimate 3000 nano ultra‐performance liquid chromatography‐tandem Q Exactive plus mass spectrometry system (LuMing Biotech, Shanghai, China).

### RNA Immunoprecipitation

RNA immunoprecipitation (RIP) assays were performed using the Magna RIP™ RNA‐Binding Protein Immunoprecipitation Kit (Millipore, Bedford, MA, USA). 4 × 10^7^ MDAH2774 cells were lysed in 200 µL Complete RIP Lysis buffer and incubated on ice for 5 min, followed by centrifugation at 14000 rpm for 15 min at 4 °C and evenly divided into 2 parts. Meanwhile, 5 µg of IgG and NONO antibody were incubated with protein A/G magnetic beads for 1 h at room temperature. Next, lysates, magnetic beads and RIP immunoprecipitation buffer were incubated overnight at 4 °C. Beads were subsequently resuspended in protein K buffer and incubated at 55 °C for 30 min. After RNA purification, the abundance of circMETTL6 were detected by qRT‐PCR.

### Co‐Immunoprecipitation (Co‐IP)

Cells were lysed in cell lysis buffer for Western and IP (Beyotime) containing 1 mM PMFS (Beyotime), followed by centrifugation at 14000 × g for 10 min at 4 °C. 2 µg of IgG, FLAG or POLR2A antibody were incubated with protein A/G magnetic beads (Bimake, Houston, TX, USA USA) for 1 h at room temperature. Equal amounts of lysate were then incubated with antibody‐beads complexes for 1 h at room temperature. Immunoprecipitates were boiled in 1× SDS loading buffer and analyzed by western blotting.

### Chromatin Immunoprecipitation (ChIP) Assay

ChIP assays were conducted using the SimpleChIP® Enzymatic Chromatin IP Kit (Magnetic Beads, Cell Signaling Technology, Danvers, MA, USA). MDAH2774 cells were crosslinked with 1% formaldehyde for 10 min at room temperature, quenched with 2.5 m glycine for 5 min, and washed with PBS. Nuclei were isolated, and chromatin was digested with micrococcal nuclease at 37 °C for 20 min, followed by sonication. Chromatin was immunoprecipitated with anti‐POLR2A (1:300) or nonspecific mouse IgG overnight at 4 °C. Immunocomplexes were incubated with 30 µL of ChIP‐Grade Protein G Magnetic Beads for 2 h at 4 °C, washed, and eluted with ChIP Elution Buffer at 65 °C for 30 min. Crosslinks were reversed with NaCl and proteinase K at 65 °C for 2 h. ChIP DNA was purified and quantified by qPCR.

### Protein Stability Assay

TOV112D and MDAH2774 cells were treated with 100 µg mL^−1^ Cycloheximide (CHX, MCE, NJ, USA) for 0, 4, and 8 h at 37 °C. DMSO (0.1%) was used as negative control. POLR2A protein level were determined by Western blotting using an anti‐POLR2A antibody.

### Dual‐Luciferase Reporter Assay

The sequence of GDF15 promoter was cloned into the pGL3‐basic vector (Promega, Madison, WI, USA). Truncated versions of the promoter (1‐99, 100–1000, 1–1000, and 1000–2000 bp) were constructed. Ovarian cancer cells were transfected with pGL3‐GDF15 and NONO siRNA in 24‐well plates. After 48 h, the cells were lysed with passive lysis buffer (Promega), and luciferase activity was measured using the Dual‐Luciferase Reporter Assay System (Promega). Firefly luciferase activity was normalized to Renilla luciferase activity.

### Survival Analysis

METTL6 expression data (FPKM) from ovarian cancer RNA‐seq in The Cancer Genome Atlas (TCGA) was retrieved via the GDC data portal (https://portal.gdc.cancer.gov/). All histological types of epithelial ovarian cancer (*n* = 408) in TCGA were included in the survival analysis. METTL6 expression was log2‐transformed for normalization before survival analysis. circMETTL6 expression in FFPE tissues from patients with ovarian cancer was scored using Basescope assay. Overall survival was defined as the time from the date of initial diagnosis to the date of death or the end of clinical follow up. While relapse‐free survival was defined as the time from the date of initial diagnosis to the first evidence of recurrence at any site or death, whichever occurred first, or to the end of clinical follow up. Patients were divided into low‐ and high‐risk groups based on median METTL6 or circMETTL6 expression. Survival distributions in different groups were visualized with Kaplan‐Meier curves, and the significance was assessed by a log‐rank test via the R package “survival.”

### Statistical Analysis

All experiments were repeated at least three times, and representative experiments are shown. Data are presented as mean ± standard deviation from at least three independent experiments. Comparisons between two groups were performed using a two‐tailed Student's t‐test, while comparisons across multiple groups employed one‐ or two‐way ANOVA. Statistical significance was set at P < 0.05. All statistical analyses were implemented using R software (version 3.5.1, http://www.R‐project.org).

### Declarations—Ethics Approval and Consent to Participate

All tissue samples used in the current study were obtained with permission at the time of diagnosis before any treatment was administered. The study protocol was approved by the Institutional Review Boards of Women's Hospital of Zhejiang University (Hangzhou, China). And all animal experiments were conducted according to protocols approved by the Institutional Animal Care and Use Committee (IACUC) of Zhejiang University.

## Conflict of Interest

The authors declare no conflict of interest.

## Author Contributions

Y.L. and P.L. conceived and designed the study; M.Y. and M.W. performed the experiments; Q.Q. performed the bioinformatics analysis; T.S. and A.L. assisted the experiments; X.L. conducted the survival analysis; J.L. prepared the RNA sequencing libraries; B.L. performed the pathological analysis of tumor tissues; T.S., W.L., and X.C. collected the tissue specimens and clinical data; M.Y., M.W., and T.S. wrote the manuscript; and Y.L. and P.L. revised the manuscript. All authors discussed and commented upon the study.

## Supporting information



Supporting Information

## Data Availability

The data that support the findings of this study are available from the corresponding author upon reasonable request.

## References

[1] S. Djebali , C. A. Davis , A. Merkel , A. Dobin , T. Lassmann , A. Mortazavi , A. Tanzer , J. Lagarde , W. Lin , F. Schlesinger , C. Xue , G. K. Marinov , J. Khatun , B. A. Williams , C. Zaleski , J. Rozowsky , M. Röder , F. Kokocinski , R. F. Abdelhamid , T. Alioto , I. Antoshechkin , M. T. Baer , N. S. Bar , P. Batut , K. Bell , I. Bell , S. Chakrabortty , X. Chen , J. Chrast , J. Curado , et al., Nature 2012, 489, 101. 22955620 10.1038/nature11233 PMC3684276

[2] E. P. Consortium , Nature 2012, 489, 57.22955616

[3] J. E. Wilusz , P. A. Sharp , Science 2013, 340, 440. 23620042 10.1126/science.1238522 PMC4063205

[4] L. L. Chen , Nat. Rev. Mol. Cell. Biol. 2016, 17, 205. 26908011 10.1038/nrm.2015.32

[5] E. Arnaiz , C. Sole , L. Manterola , L. Iparraguirre , D. Otaegui , C. H. Lawrie , Semin. Cancer. Biol. 2019, 58, 90. 30550956 10.1016/j.semcancer.2018.12.002

[6] L. S. Kristensen , T. Jakobsen , H. Hager , J. Kjems , Nat. Rev. Clin. Oncol. 2022, 19, 188. 34912049 10.1038/s41571-021-00585-y

[7] L. Fang , W. W. Du , F. M. Awan , J. Dong , B. B. Yang , Cancer. Lett. 2019, 459, 216. 31199987 10.1016/j.canlet.2019.05.036

[8] F. Yang , A. Hu , D. Li , J. Wang , Y. Guo , Y. Liu , H. Li , Y. Chen , X. Wang , K. Huang , L. Zheng , Q. Tong , Mol. Cancer. 2019, 18, 158. 31718709 10.1186/s12943-019-1094-z PMC6852727

[9] Y. Hong , H. Qin , Y. Li , Y. Zhang , X. Zhuang , L. Liu , K. Lu , L. Li , X. Deng , F. Liu , S. Shi , G. Liu , J. Cell. Physiol. 2019, 234, 19895. 30963578 10.1002/jcp.28588 PMC6766960

[10] H. Sung , J. Ferlay , R. L. Siegel , M. Laversanne , I. Soerjomataram , A. Jemal , F. Bray , CA Cancer. J. Clin. 2021, 71, 209. 33538338 10.3322/caac.21660

[11] Cancer Genome Atlas Research Network , Nature 2011, 474, 609.21720365

[12] I. Vergote , A. González‐Martín , I. Ray‐Coquard , P. Harter , N. Colombo , P. Pujol , D. Lorusso , M. R. Mirza , B. Brasiuniene , R. Madry , J. D. Brenton , M. G. E. M. Ausems , R. Büttner , D. Lambrechts , I. Vergote , M. Ausems , B. Brasiuniene , J. Brenton , R. Büttner , N. Colombo , A. González‐Martín , P. Harter , D. Lambrechts , D. Lorusso , R. Madry , M. R. Mirza , P. Pujol , I. Ray‐Coquard , M. Abreu , S. Balboni , et al., Ann. Oncol. 2022, 33, 276.34861371

[13] C. Fumagalli , I. Betella , A. Rappa , M. di Giminiani , M. Gaiano , L. A. De Vitis , B. Zambetti , D. Vacirca , F. Multinu , K. Venetis , N. Colombo , M. Barberis , E. G. Rocco , Cancers 2022, 14, 1638. 35406410 10.3390/cancers14071638 PMC8996829

[14] D. K. Armstrong , R. D. Alvarez , F. J. Backes , J. N. Bakkum‐Gamez , L. Barroilhet , K. Behbakht , A. Berchuck , L.‐M. Chen , V. C. Chitiyo , M. Cristea , M. DeRosa , E. L. Eisenhauer , D. M. Gershenson , H. J. Gray , R. Grisham , A. Hakam , A. Jain , A. Karam , G. E. Konecny , C. A. Leath III , G. Leiserowitz , J. Liu , L. Martin , D. Matei , M. McHale , K. McLean , D. S. Miller , S. Percac‐Lima , S. W. Remmenga , J. Schorge , et al., J. Natl. Compr. Cancer Network 2022, 20, 972. 10.6004/jnccn.2022.0047 36075393

[15] V. V. Ignatova , S. Kaiser , J. S. Y. Ho , X. Bing , P. Stolz , Y. X. Tan , C. L. Lee , F. P. H. Gay , P. R. Lastres , R. Gerlini , B. Rathkolb , A. Aguilar‐Pimentel , A. Sanz‐Moreno , T. Klein‐Rodewald , J. Calzada‐Wack , E. Ibragimov , M. Valenta , S. Lukauskas , A. Pavesi , S. Marschall , S. Leuchtenberger , H. Fuchs , V. Gailus‐Durner , M. H. de Angelis , S. Bultmann , O. J. Rando , E. Guccione , S. M. Kellner , R. Schneider , Sci. Adv. 2020, 6, eaaz4551. 32923617 10.1126/sciadv.aaz4551 PMC7449687

[16] R. Chen , J. Zhou , L. Liu , X. L. Mao , X. Zhou , W. Xie , Commun. Biol. 2021, 4, 1361. 34862464 10.1038/s42003-021-02890-9 PMC8642396

[17] A. Bolatkan , K. Asada , S. Kaneko , K. Suvarna , N. Ikawa , H. Machino , M. Komatsu , S. Shiina , R. Hamamoto , Int. J. Oncol. 2022, 60, 4. 34913069 10.3892/ijo.2021.5294 PMC8698744

[18] M. Wu , Q. Qiu , Q. Zhou , J. Li , J. Yang , C. Zheng , A. Luo , X. Li , H. Zhang , X. Cheng , W. Lu , P. Liu , B. Lu , Y. Lu , Mol. Cancer. 2022, 21, 137. 35768865 10.1186/s12943-022-01611-y PMC9241180

[19] R. Xie , Xu. Chen , L. Cheng , M. Huang , Q. Zhou , J. Zhang , Y. Chen , S. Peng , Z. Chen , W. Dong , J. Huang , T. Lin , Mol. Ther. 2021, 29, 291. 32950106 10.1016/j.ymthe.2020.08.018 PMC7791011

[20] P. Feng , L. Li , T. Deng , Y. Liu , N. Ling , S. Qiu , L. Zhang , Bo. Peng , W. Xiong , L. Cao , L. Zhang , M. Ye , J. Cell. Mol. Med. 2020, 24, 4368. 32168434 10.1111/jcmm.15141 PMC7176863

[21] S.‐J. Kim , J.‐S. Ju , M.‐H. Kang , Ji. E. Won , Y. Ha. Kim , P. V. Raninga , K. K. Khanna , B. Gyorffy , C‐Gi. Pack , H.‐D. Han , H. J. Lee , G. Gong , Y. Shin , G. B. Mills , S‐Il. Eyun , Y.‐Y. Park , Theranostics 2020, 10, 7974. 32724453 10.7150/thno.45037 PMC7381744

[22] Y. Wei , H. Luo , P. P. Yee , L. Zhang , Z. Liu , H. Zheng , L. Zhang , B. Anderson , M. Tang , S. Huang , W. Li , Adv. Sci. 2021, 8, e2102653. 10.1002/advs.202102653 PMC8693076 34716691

[23] Z. Hu , L. Dong , S. Li , Z. Li , Y. Qiao , Y. Li , J. Ding , Z. Chen , Y. Wu , Z. Wang , S. Huang , Q. Gao , Y. Zhao , X. He , Hepatology 2020, 72, 548. 31815296 10.1002/hep.31062

[24] P. A. Rudnick , S. P. Markey , J. Roth , Y. Mirokhin , X. Yan , D. V. Tchekhovskoi , N. J. Edwards , R. R. Thangudu , K. A. Ketchum , C. R. Kinsinger , M. Mesri , H. Rodriguez , S. E. Stein , J. Proteome. Res. 2016, 15, 1023. 26860878 10.1021/acs.jproteome.5b01091 PMC5117628

[25] X. Li , M. Chen , B. Liu , P. Lu , X. Lv , X. Zhao , S. Cui , P. Xu , Y. Nakamura , R. Kurita , B. Chen , D. C. S. Huang , De‐P. Liu , M. Liu , Q. Zhao , Nucleic Acids Res. 2021, 49, 9711. 34379783 10.1093/nar/gkab671 PMC8464040

[26] S. Chen , J. Wang , K. Zhang , B. Ma , X. Li , R. Wei , H. Nian , Cell. Death. Dis. 2023, 14, 610. 37716986 10.1038/s41419-023-06132-0 PMC10505237

[27] A. Emili , M. Shales , S. McCracken , W. Xie , P. W. Tucker , R. Kobayashi , B. J. Blencowe , C. J. Ingles , RNA 2002, 8, 1102. 12358429 10.1017/s1355838202025037 PMC1370324

[28] D. Yanling Zhao , G. Gish , U. Braunschweig , Y. Li , Z. Ni , F. W. Schmitges , G. Zhong , Ke. Liu , W. Li , J. Moffat , M. Vedadi , J. Min , T. J. Pawson , B. J. Blencowe , J. F. Greenblatt , Nature 2016, 529. 10.1038/nature16469 26700805

[29] X. Wang , S. J. Baek , T. E. Eling , Biochem. Pharmacol. 2013, 85, 597. 23220538 10.1016/j.bcp.2012.11.025 PMC3566326

[30] A. R. Bauskin , H.‐P. Zhang , W. D. Fairlie , X. Y. He , P. K. Russell , A. G. Moore , D. A. Brown , K. K. Stanley , S. N. Breit , EMBO J. 2000, 19, 2212. 10811612 10.1093/emboj/19.10.2212 PMC384362

[31] A. R. Bauskin , D. A. Brown , S. Junankar , K. K. Rasiah , S. Eggleton , M. Hunter , T. Liu , D. Smith , T. Kuffner , G. J. Pankhurst , H. Johnen , P. J. Russell , W. Barret , P. D. Stricker , J. J. Grygiel , J. G. Kench , S. M. Henshall , R. L. Sutherland , S. N. Breit , Cancer Res. 2005, 65, 2330. 15781647 10.1158/0008-5472.CAN-04-3827

[32] S. Li , Y. M. Ma , P. S. Zheng , P. Zhang , J. Exp. Clin. Cancer. Res. 2018, 37, 80. 29636108 10.1186/s13046-018-0744-0 PMC5894198

[33] R. S. Mehta , M. Song , N. Bezawada , K. Wu , X. Garcia‐Albeniz , T. Morikawa , C. S. Fuchs , S. Ogino , E. L. Giovannucci , A. T. Chan , J. Natl. Cancer. Inst. 2014, 106, dju016. 24565956 10.1093/jnci/dju016 PMC3982884

[34] A. C. Staff , J. Trovik , E. A. G. Zahl , E. Wik , K. C. Wollert , T. Kempf , H. B. Salvesen , Clin. Cancer. Res. 2011, 17, 4825. 21616994 10.1158/1078-0432.CCR-11-0715

[35] S. Ghafouri‐Fard , T. Khoshbakht , B. M. Hussen , M. Taheri , M. Samsami , Cancer. Cell Int. 2022, 22, 172. 35488239 10.1186/s12935-022-02602-1 PMC9052556

[36] L. Ge , Yu. Sun , Y. Shi , G. Liu , F. Teng , Z. Geng , X. Chen , H. Xu , J. Xu , X. Jia , J. Ovarian. Res. 2022, 15, 58. 35550610 10.1186/s13048-022-00988-0 PMC9097182

[37] X. Wang , T. Chen , C. Li , W. Li , X. Zhou , Y. Li , D. Luo , N. Zhang , B. Chen , L. Wang , W. Zhao , S. Fu , Q. Yang , J. Hematol. Oncol. 2022, 15, 122. 36038948 10.1186/s13045-022-01345-w PMC9425971

[38] G. Benegiamo , L. S. Mure , G. Erikson , H. D. Le , E. Moriggi , S. A. Brown , S. Panda , Cell Metab. 2018, 27, 404. 29358041 10.1016/j.cmet.2017.12.010 PMC6996513

[39] S. P. Yadav , H. Hao , H.‐J. Yang , M‐A. I. Kautzmann , M. Brooks , J. Nellissery , B. Klocke , M. Seifert , A. Swaroop , Hum. Mol. Genet. 2014, 23, 2132. 24301678 10.1093/hmg/ddt609 PMC3959818

[40] C. L. Bladen , D. Udayakumar , Y. Takeda , W. S. Dynan , J. Biol. Chem. 2005, 280, 5205. 15590677 10.1074/jbc.M412758200

[41] X. Nie , Q. Xu , C. Xu , F. Chen , Q. Wang , D. Qin , R. Wang , Z. Gao , X. Lu , X. Yang , Yu. Wu , C. Gu , W. Xie , L. Li , Nat. Commun. 2023, 14, 4275. 37460529 10.1038/s41467-023-39924-1 PMC10352294

[42] L. Fang , F. Li , C. Gu , Curr. Pharm. Des. 2019, 25, 654. 30947652 10.2174/1381612825666190402101143

[43] D. Kim , G. Pertea , C. Trapnell , H. Pimentel , R. Kelley , S. L. Salzberg , Genome Biol. 2013, 14, R36. 23618408 10.1186/gb-2013-14-4-r36 PMC4053844

[44] X.‐Ou. Zhang , R. Dong , Y. Zhang , J.‐L. Zhang , Z. Luo , J. Zhang , L.‐L. Chen , Li. Yang , Genome Res. 2016, 26, 1277. 27365365 10.1101/gr.202895.115 PMC5052039

[45] A. T. Sengal , A.‐M. Patch , C. E. Snell , D. S. Smith , S. C. Y. Leung , A. Talhouk , E. D. Williams , J. N. McAlpine , P. M. Pollock , Clin. Cancer. Res. 2020, 26, 4569. 32414751 10.1158/1078-0432.CCR-19-4088

[46] A.‐M. Baker , W. Huang , X.‐M. M. Wang , M. Jansen , X.‐J. Ma , J. Kim , C. M. Anderson , X. Wu , L. Pan , N. Su , Y. Luo , E. Domingo , T. Heide , A. Sottoriva , A. Lewis , A. D. Beggs , N. A. Wright , M. Rodriguez‐Justo , E. Park , I. Tomlinson , T. A. Graham , Nat. Commun. 2017, 8, 1998. 29222441 10.1038/s41467-017-02295-5 PMC5722928

[47] L. Zhang , Q. Zhou , Q. Qiu , L. Hou , M. Wu , J. Li , X. Li , B. Lu , X. Cheng , P. Liu , W. Lu , Y. Lu , Mol. Cancer. 2019, 18, 144. 31623606 10.1186/s12943-019-1080-5 PMC6796346

[48] S. Kovaka , A. V. Zimin , G. M. Pertea , R. Razaghi , S. L. Salzberg , M. Pertea , Genome Biol. 2019, 20, 278. 31842956 10.1186/s13059-019-1910-1 PMC6912988

[49] M. I. Love , W. Huber , S. Anders , Genome Biol. 2014, 15, 550. 25516281 10.1186/s13059-014-0550-8 PMC4302049

