# Supplementary material for: CircMETTL6 Suppresses Ovarian Cancer Cell Growth and Metastasis Through Inhibition of GDF15 Transcription by Disrupting the NONO‐POLR2A Complex
Source: Adv Sci (Weinh). 2025 Feb 3;12(12):2411717. doi: 10.1002/advs.202411717 (PMC11948058; doi:10.1002/advs.202411717)
Supplement: Supplementary file 1 — Supporting Information [file ADVS-12-2411717-s001.docx]

**CircMETTL6 Suppresses Ovarian Cancer Cell Growth and Metastasis Through Inhibition of GDF15 Transcription by Disrupting the NONO-POLR2A Complex**

Mengqian Yu *et al.*

**Supporting Information**

**Table S1** Top ten differentially expressed circRNAs in RNA-seq data


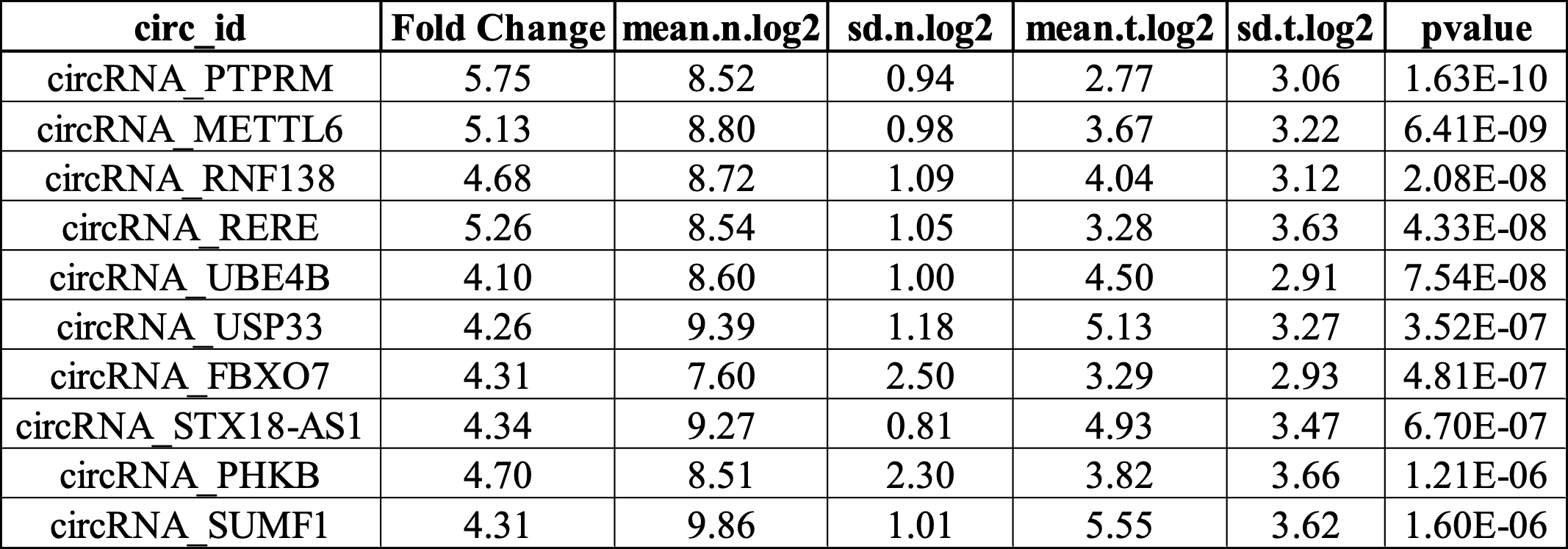


**Table S2** Proteins identified by mass spectrometry analysis of RNA pull-down fractions


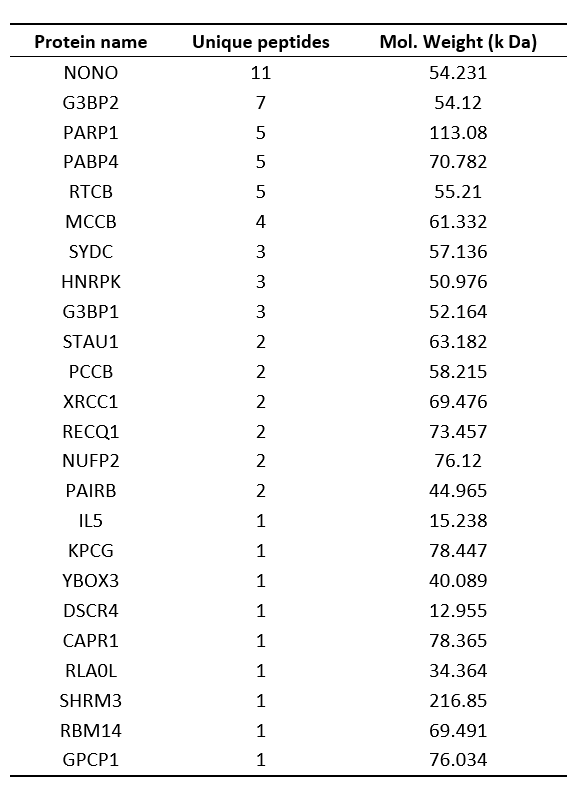


Note: Proteins of 50-70 kD and only present in the sense fraction with multiple detected peptides were selected for subsequent validation experiments.

**Table S3** Sequences of primers and siRNAs used in the study

| Oligo ids | Sequence(5’-3’) |
| --- | --- |
| GAPDH F | AGCTCACTGGCATGGCCTTC |
| GADPH R | CGCCTGCTTCACCACCTTCT |
| β-actin F | GGACTTCGAGCAAGAGATGG |
| β-actin R | AGCACTGTGTTGGCGTACAG |
| CircMETTL6 F | GTGTGCCAAGAGTTTTCCTTCAGAG |
| CircMETTL6 R | AACATCCACAGACTCTGGCGGT |
| METTL6 F | CAGGCAAGGATTCTCACCTCT |
| METTL6 R | TGGTCCAGTGTCTGTCTTTGA |
| METTL6(exon) F | CAAAATCCTTTATATGATACAGAAAG |
| METTL6(exon) R | CTCATGAAAGGTCAGGACTTAGGATC |
| CircMETTL6 full length F1 | CCAGAGTCTGTGGATGTTGTTA |
| CircMETTL6 full length R1 | CGGTACATGATCCAGAAGATCATC |
| CircMETTL6 full length F2 | CATGAGCAAAATCCTTTATATGA |
| CircMETTL6 full length R2 | AAAGGTCAGGACTTAGGATC |
| NONO F | CTAGCGGAGATTGCCAAAGTG |
| NONO R | GTTCGTTGGACACATACTGAGG |
| POLR2A F | GAGCGGGGTGAAGTGATGAA |
| POLR2A R | GCTTGTAAGGGCCACTGTCT |
| NONO FL-Flag F | GGATCTATTTCCGGTGAATTCATGCAGAGTAATAAAACTTTTAACTTGGA |
| NONO FL-Flag R | GGAGGGAGAGGGGCGGGATCCTTATTTATCGTCGTCATCCTTATAATCG |
| △RRM1 F | AGAAGACCTTCACCCAACGACGCTTTGCCTGCCATAGTG |
| △RRM1 R | TCGTTGGGTGAAGGTCTTCTCT |
| △RRM2 F | CGCTTTGCCTGCCATAGTTTAGATGATGAAGAGGGACTTCCAG |
| △RRM2 R | AAACTATGGCAGGCAAAGCGC |
| △NOPS F | ATGGACCAGGCCATGCGCTGGAAGGCA |
| △NOPS R | CAGCGCATGGCCTGGTCCATGGGCTCCACA |
| △CC F | TATACCTTCCCTGATGCGAGAGAG |
| △CC R | CTCGCATCAGGGAAGGTATATTCATACTCAAAGGAGCCAGGC |
| GDF15 promoter F | CGCTCGAGCTCGGGGCCTGCAGGAAGTTT |
| GDF15 promoter R | CGAAGCTTTGAACCGCACTGACACCTGGG |
| GDF15 F | GACCCTCAGAGTTGCACTCC |
| GDF15 R | GCCTGGTTAGCAGGTCCTC |
| Negative Control siRNA | UUCUCCGAACGUGUCACGUTT |
| NONO siRNA1 | CAAACGUCGCCGAUACUAATT |
| NONO siRNA2 | GAUGGAAGCUGCACGCCAUTT |
| POLR2A siRNA1 | CCUGGUGAAGACAAUGAAATT |
| POLR2A siRNA2 | GCGGCAGACGUUUGAGAAUTT |
| GDF15 siRNA1 | CCAAACAGCUGUAUUUAUATT |
| GDF15 siRNA2 | GUCUGAUGGAACUGUGUAUTT |

**Table S4** Demographic and clinical characteristics of ovarian cancer patients used in quantitative real-time PCR (qRT-PCR) assays


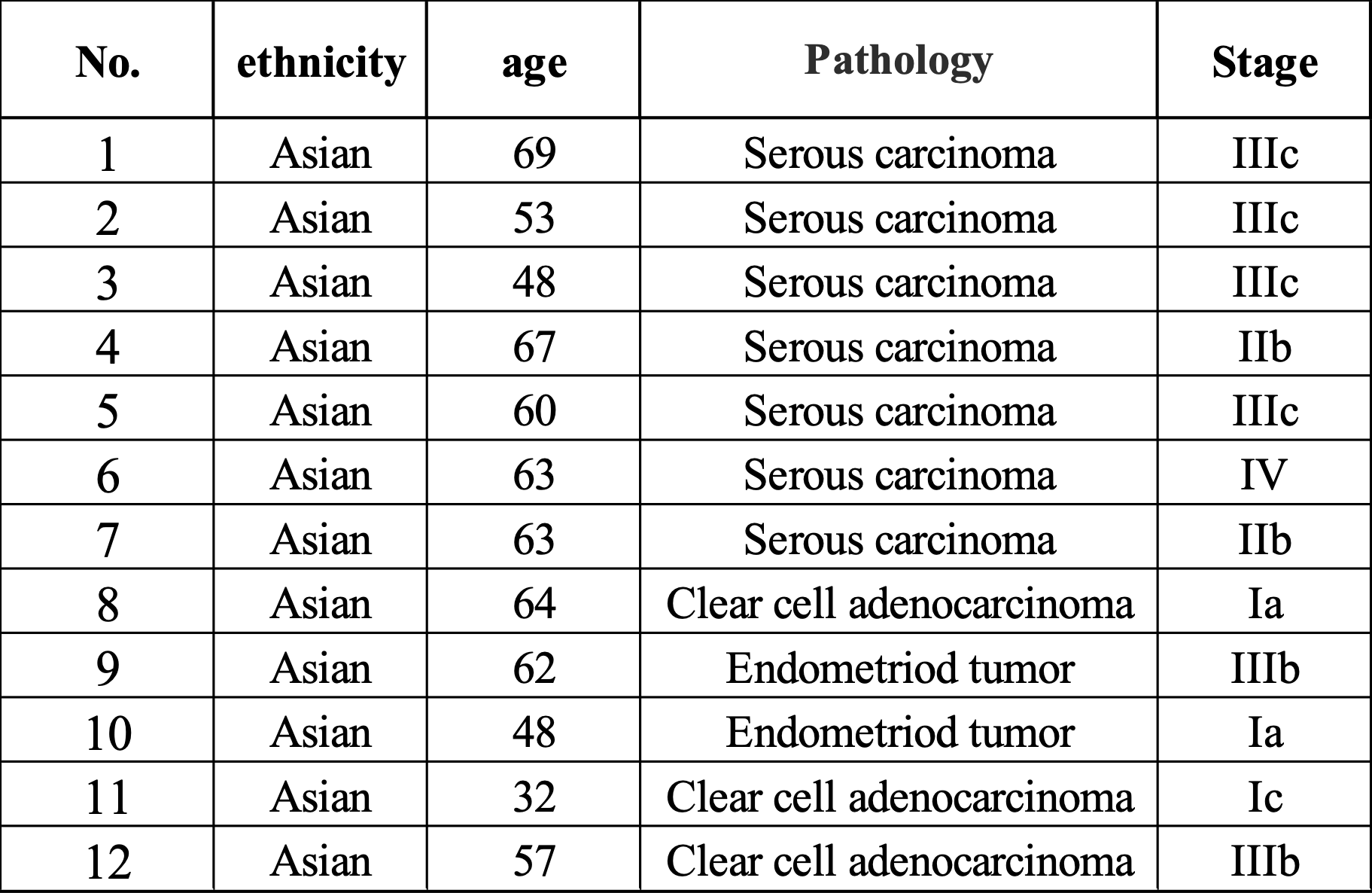


**Table S5** Information of antibodies used in the study

| **Antibodies** | **Company** | **Catelogue#** | **Species** |
| --- | --- | --- | --- |
| GAPDH | Proteintech | 60004-1-Ig | mouse |
| β-actin | Proteintech | 66009-1-Ig | mouse |
| METTL6 | Proteintech | 16527-1-AP | rabbit |
| NONO | Santa Cruz | sc-376865 | mouse |
| GDF15 | Santa Cruz | sc-377195 | mouse |
| FLAG | sigma | A2220 | mouse |
| POLR2A | BioLegend | 664906 | mouse |
| SAPCD2 | Abisn | abs105974 | rabbit |
| Anti-rabbit IgG, HRP-linked Antibody | Cell Signaling Technology | 7074 |  |
| Anti-mouse IgG, HRP-linked Antibody | Cell Signaling Technology | 7076 |  |

**Table S6** Demographic and clinical characteristics of ovarian cancer patients used in Basescope assays


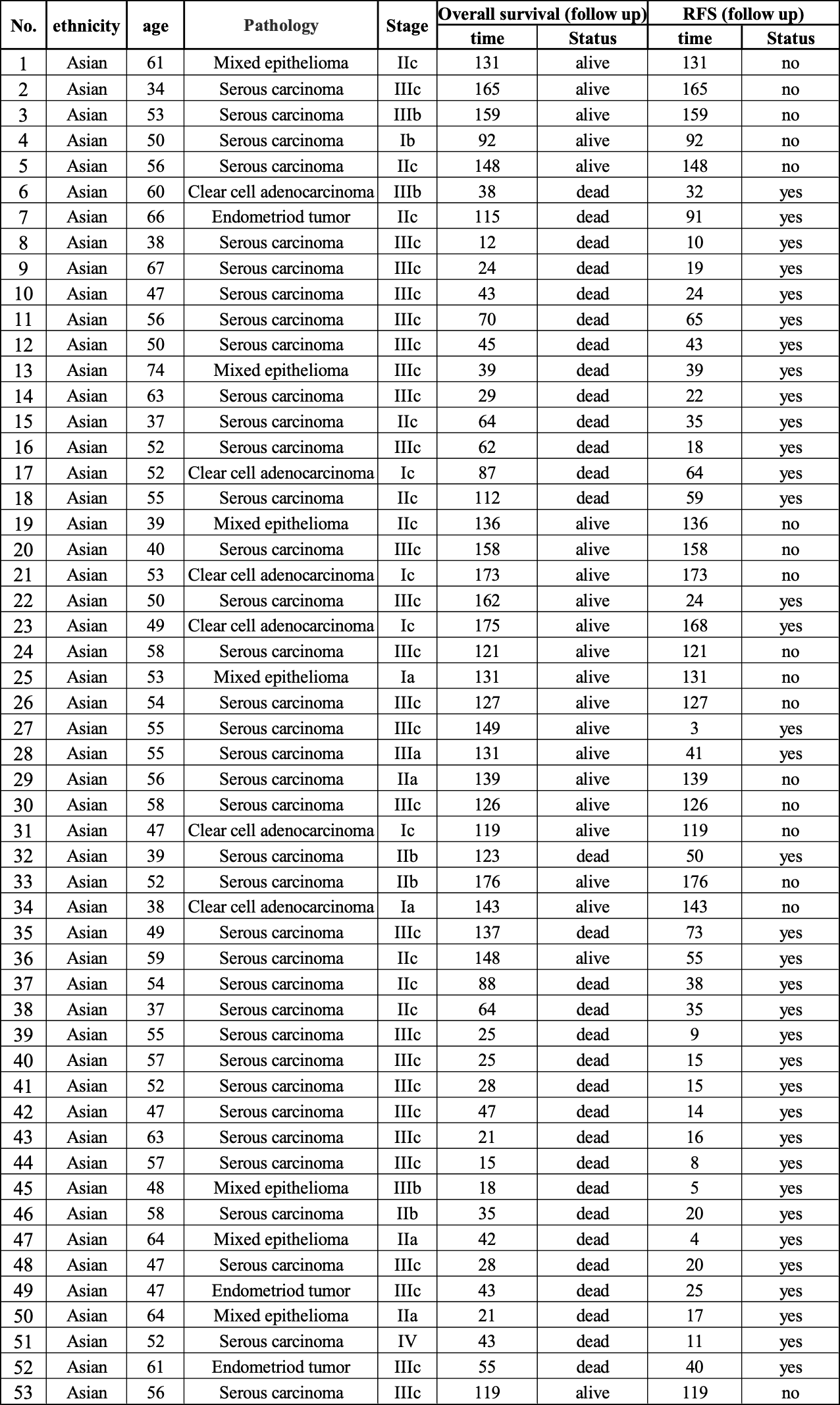


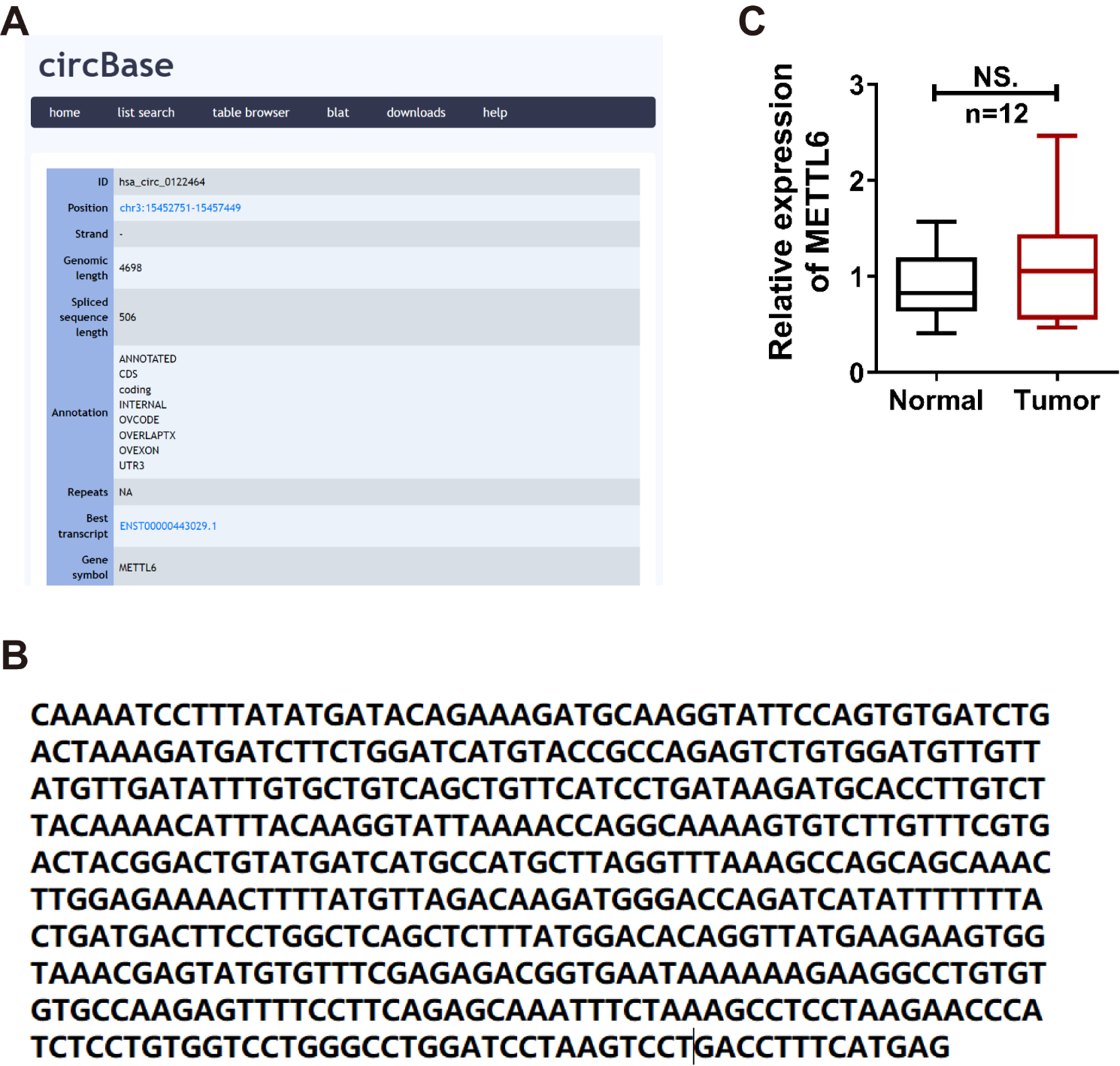


**Fig. S1 (related to Figure 1). circMETTL6 is downregulated in ovarian cancer.**

(**A**) circBase annotation of circMETTL6 (ID: hsa_circ_0122464).

(**B**) Full-length sequence of circMETTL6 confirmed by Sanger sequencing.

(**C**) qRT-PCR analysis of METTL6 expression in an independent cohort consisting of 12 tumor tissues from ovarian cancer patients and normal ovarian tissues from patients with benign gynecological diseases. NS: no significance.


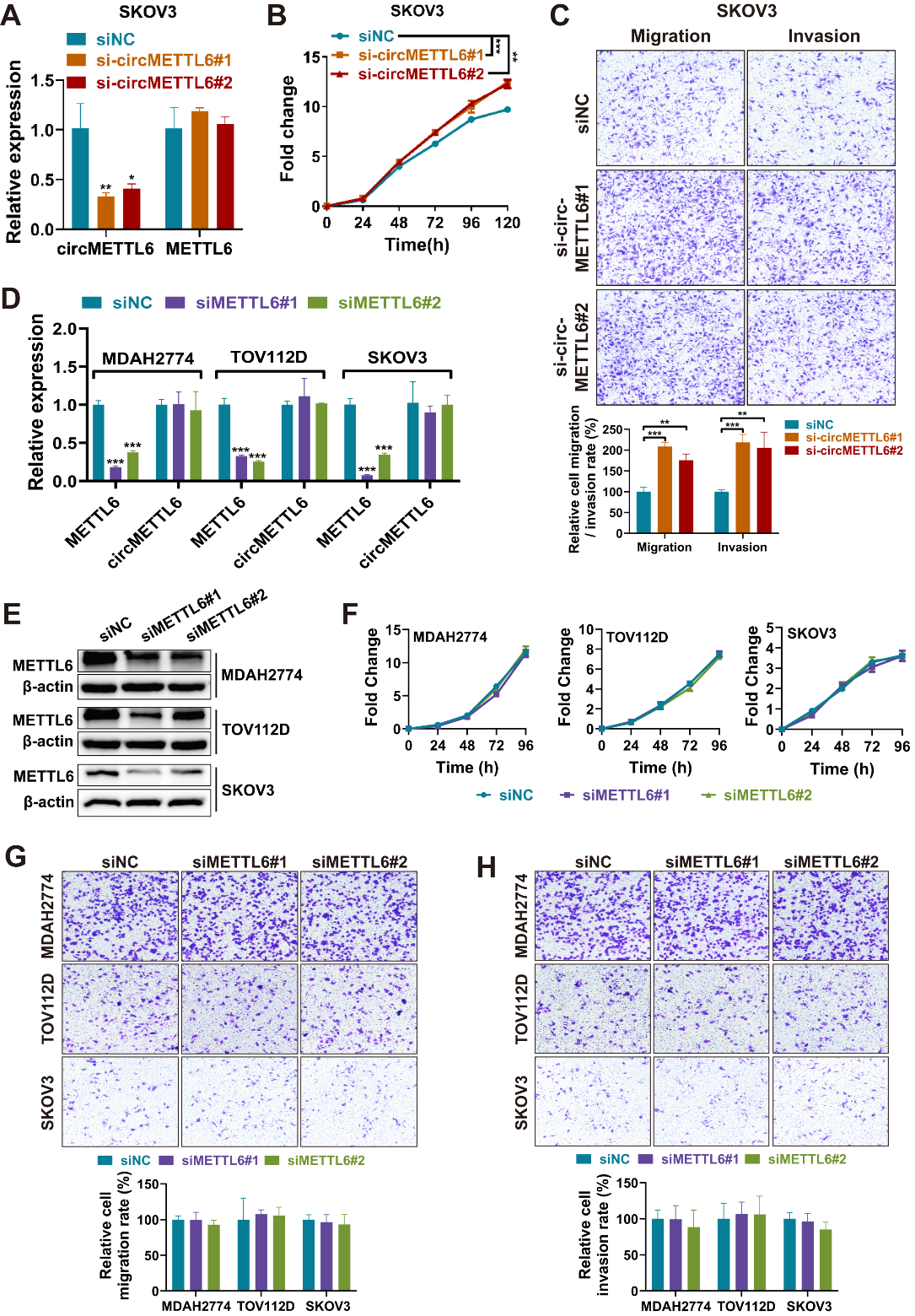


**Fig. S2 (related to Figure 2). circMETTL6 plays a tumor suppressive role in ovarian cancer cells.**

(**A**) qRT-PCR analysis of circMETTL6 and METTL6 expression in SKOV3 cells transfected with either circMETTL6 siRNAs or negative control siRNA (siNC). Two siRNAs (si-circMETTL6#1 and si-circMETTL6#2) were designed for targeting circMETTL6.

(**B**) Cell Counting Kit-8 (CCK-8) assays in SKOV3 cells transfected with either circMETTL6 or NC siRNAs.

(**C**) Migration and invasion abilities of SKOV3 cells transfected with circMETTL6 or NC siRNAs, assessed by transwell assays. Scale bars, 100 μm. Statistical analysis is shown in the lower panel.

(**D**) qRT-PCR analysis of METTL6 and circMETTL6 expression in ovarian cancer cells transfected with either METTL6 siRNAs or negative control siRNA (siNC). Two siRNAs (si-METTL6#1 and si-METTL6#2) were designed for targeting METTL6.

(**E**) Western blot analysis of METTL6 protein expression in ovarian cancer cells transfected with either METTL6 or NC siRNAs.

(**F, G, H**) CCK-8 (**F**), migration (**G**) and invasion (**H**) assays for ovarian cancer cells transfected with either METTL6 or NC siRNAs. For (**G**) and (**H**), statistical analysis is shown in the lower panel. Scale bars, 100 μm.

Data were presented as mean ± standard deviation (SD); n = 3; Statistical significance was determined using Student’s two-sided t-test; *P < 0.05, **P < 0.01, ***P < 0.001. These descriptions are not reiterated in the subsequent figure legends.


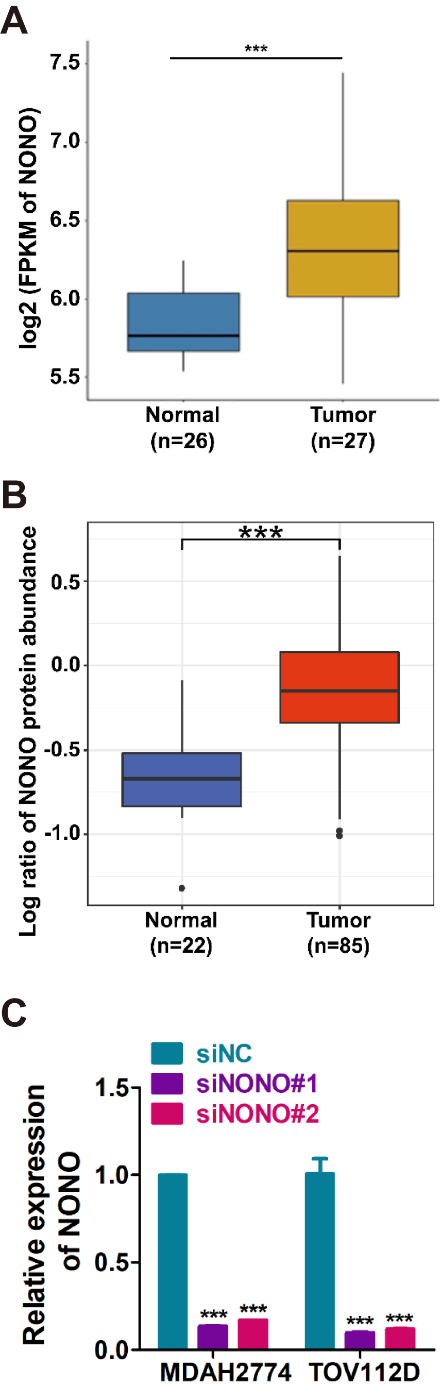


**Fig. S3 (related to Figure 3). circMETTL6 physically interacts with the oncogenic protein NONO.**

(**A**) mRNA expression of NONO in primary ovarian tumors (n=27) and normal ovarian tissues (n=26) based on our RNA-seq data.

(**B**) Protein expression of NONO in primary ovarian tumors (n=85) and normal ovarian tissues (n=22) analyzed using data from the Clinical Proteomic Tumor Analysis Consortium (CPTAC).

(**C**) qRT-PCR analysis of NONO mRNA expression in ovarian cancer cells transfected with either NONO siRNA or negative control siRNA (siNC). Two siRNAs (siNONO#1 and siNONO#2) were designed for targeting NONO.


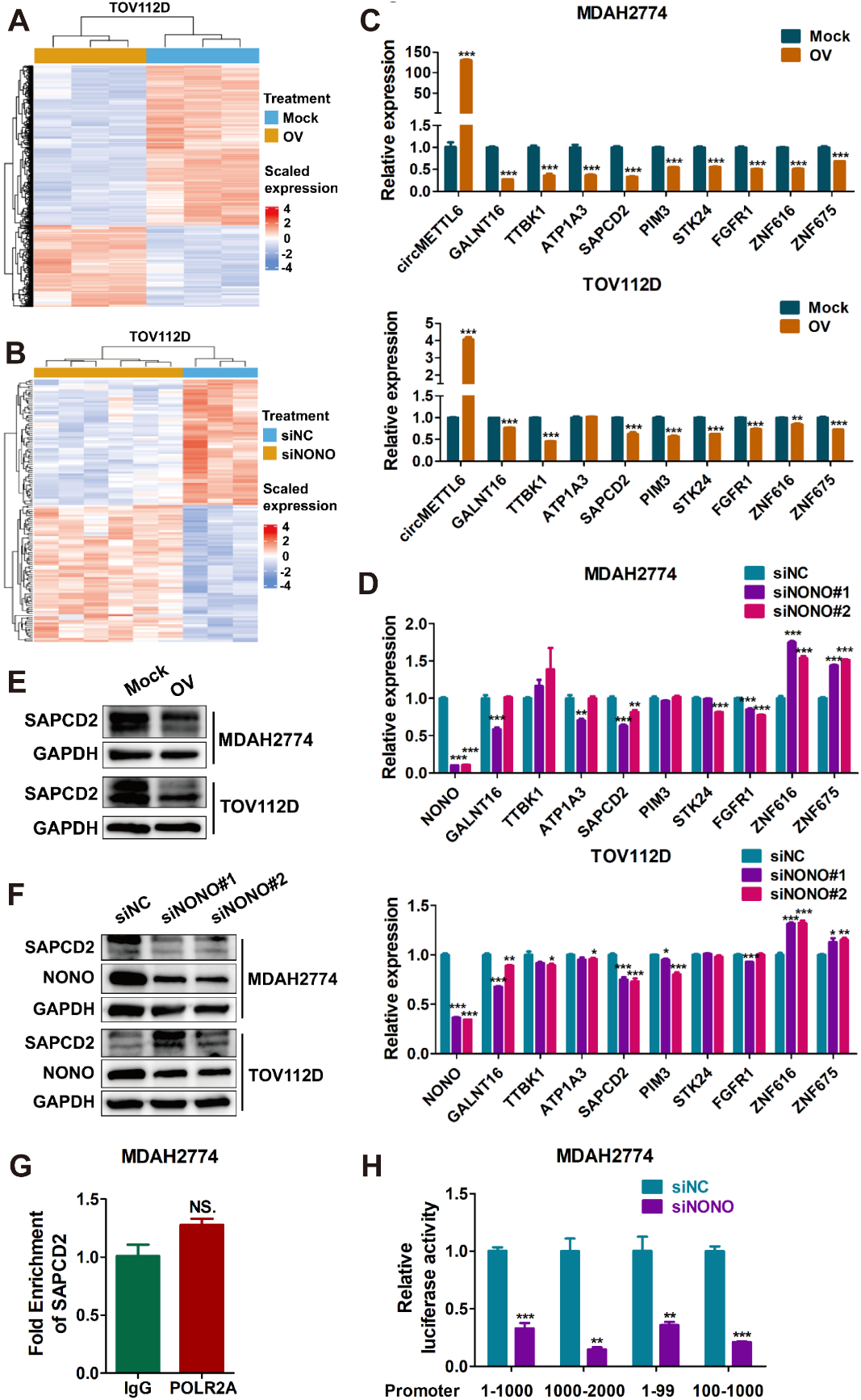


**Fig. S4, (related to Figure 5). circMETTL6 disrupts NONO-POLR2A complex to suppress GDF15 transcription.**

(**A**) Hierarchical clustering of differentially expressed genes between control (Mock) and circMETTL6-overexpressing (OV) TOV112D cells.

(**B**) Hierarchical clustering of differentially expressed genes between control (siNC) and NONO-silenced (siNONO#1 and siNONO#2) TOV112D cells.

(**C**) qRT-PCR analysis of differentially expressed genes between circMETTL6-overexpressing and control ovarian cancer cells (MDAH2774 and TOV112D).

(**D**) qRT-PCR analysis of differentially expressed genes between NONO-silenced and control ovarian cancer cells (MDAH2774 and TOV112D).

(**E**) Western blot analysis of SAPCD2 in MDAH2774 and TOV112D cells with control (Mock) and circMETTL6-overexpression (OV).

(**F**) Western blot analysis of SAPCD2 in MDAH2774 and TOV112D cells with control (siNC) and NONO knockdown.

(**G**) ChIP-qPCR analysis of the binding of POLR2A with SAPCD2 promoter in MDAH2774 cells. An anti-IgG or anti-POLR2A antibody was used. NS: no significant.

(**H**) Dual-luciferase reporter assays measuring transcription levels of GDF15 in NONO knockdown and control MDAH2774 cells with various GDF15 promoter truncators.


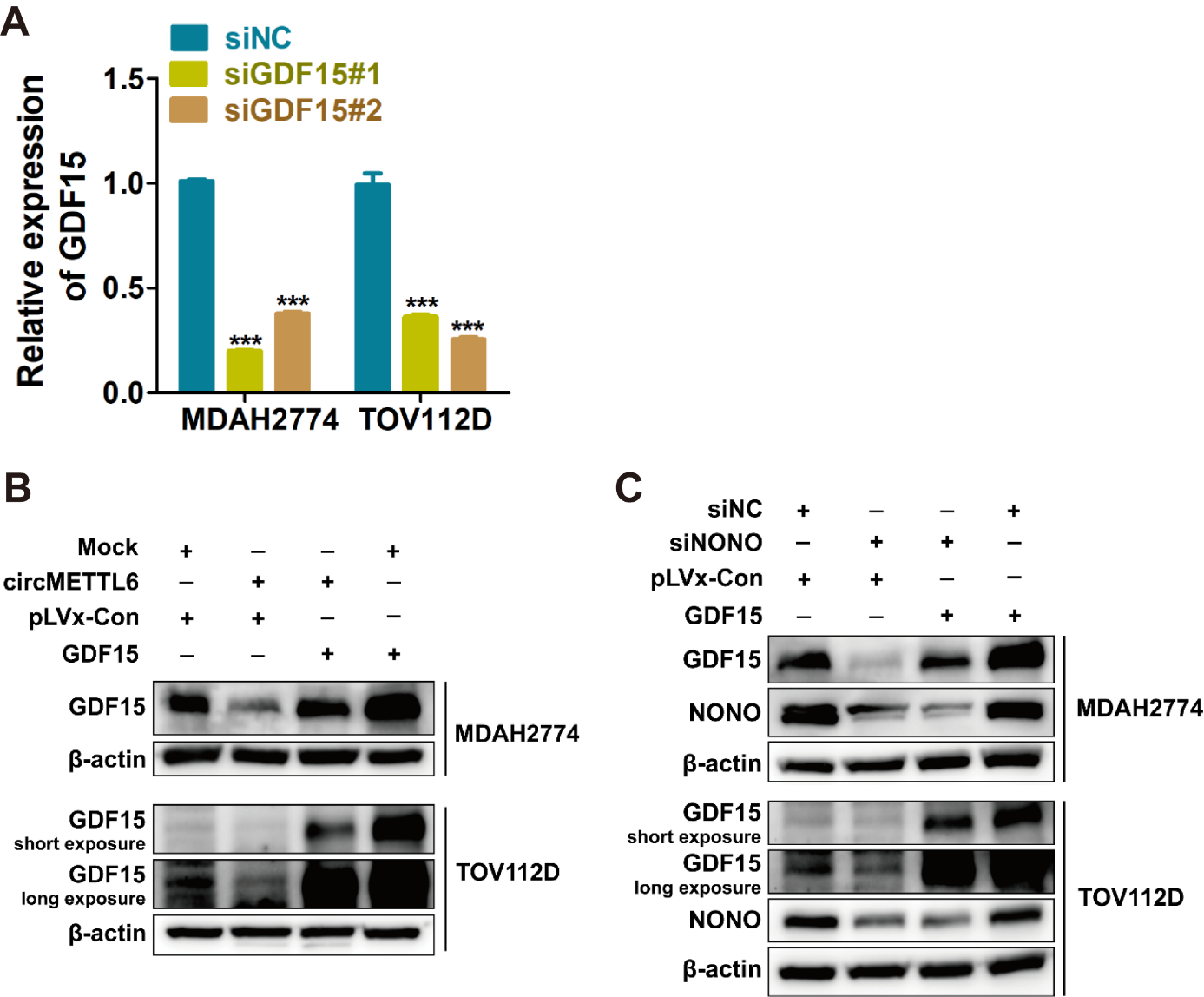


**Fig. S5, (related to Figure 6). circMETTL6 inhibits ovarian cancer cells proliferation and migration by downregulating GDF15 expression.**

(**A**) qRT-PCR analysis of GDF15 mRNA levels in ovarian cancer cells transfected with GDF15-specific siRNAs (siGDF15#1 and siGDF15#2) or negative control siRNA (siNC).

(**B**) Western blot analysis of GDF15 expression in circMETTL6-overexpressing ovarian cancer cells transfected with GDF15 overexpression or control plasmids (pLVx-Con).

(**C**) Western blot analysis of GDF15 expression in NONO-silenced ovarian cancer cells transfected with GDF15 overexpression or control plasmids.
